# A search for new phenomena in pp collisions at $\sqrt{s} = 13\,\text {TeV}$ in final states with missing transverse momentum and at least one jet using the $\alpha _{\mathrm {T}}$ variable

**DOI:** 10.1140/epjc/s10052-017-4787-8

**Published:** 2017-05-08

**Authors:** V. Khachatryan, A. M. Sirunyan, A. Tumasyan, W. Adam, E. Asilar, T. Bergauer, J. Brandstetter, E. Brondolin, M. Dragicevic, J. Erö, M. Flechl, M. Friedl, R. Frühwirth, V. M. Ghete, C. Hartl, N. Hörmann, J. Hrubec, M. Jeitler, A. König, I. Krätschmer, D. Liko, T. Matsushita, I. Mikulec, D. Rabady, N. Rad, B. Rahbaran, H. Rohringer, J. Schieck, J. Strauss, W. Waltenberger, C.-E. Wulz, O. Dvornikov, V. Makarenko, V. Zykunov, V. Mossolov, N. Shumeiko, J. Suarez Gonzalez, S. Alderweireldt, E. A. De Wolf, X. Janssen, J. Lauwers, M. Van De Klundert, H. Van Haevermaet, P. Van Mechelen, N. Van Remortel, A. Van Spilbeeck, S. Abu Zeid, F. Blekman, J. D’Hondt, N. Daci, I. De Bruyn, K. Deroover, S. Lowette, S. Moortgat, L. Moreels, A. Olbrechts, Q. Python, S. Tavernier, W. Van Doninck, P. Van Mulders, I. Van Parijs, H. Brun, B. Clerbaux, G. De Lentdecker, H. Delannoy, G. Fasanella, L. Favart, R. Goldouzian, A. Grebenyuk, G. Karapostoli, T. Lenzi, A. Léonard, J. Luetic, T. Maerschalk, A. Marinov, A. Randle-conde, T. Seva, C. Vander Velde, P. Vanlaer, D. Vannerom, R. Yonamine, F. Zenoni, F. Zhang, A. Cimmino, T. Cornelis, D. Dobur, A. Fagot, G. Garcia, M. Gul, I. Khvastunov, D. Poyraz, S. Salva, R. Schöfbeck, A. Sharma, M. Tytgat, W. Van Driessche, E. Yazgan, N. Zaganidis, H. Bakhshiansohi, C. Beluffi, O. Bondu, S. Brochet, G. Bruno, A. Caudron, S. De Visscher, C. Delaere, M. Delcourt, B. Francois, A. Giammanco, A. Jafari, P. Jez, M. Komm, G. Krintiras, V. Lemaitre, A. Magitteri, A. Mertens, M. Musich, C. Nuttens, K. Piotrzkowski, L. Quertenmont, M. Selvaggi, M. Vidal Marono, S. Wertz, N. Beliy, W. L. Aldá Júnior, F. L. Alves, G. A. Alves, L. Brito, C. Hensel, A. Moraes, M. E. Pol, P. Rebello Teles, E. Belchior Batista Das Chagas, W. Carvalho, J. Chinellato, A. Custódio, E. M. Da Costa, G. G. Da Silveira, D. De Jesus Damiao, C. De Oliveira Martins, S. Fonseca De Souza, L. M. Huertas Guativa, H. Malbouisson, D. Matos Figueiredo, C. Mora Herrera, L. Mundim, H. Nogima, W. L. Prado Da Silva, A. Santoro, A. Sznajder, E. J. Tonelli Manganote, A. Vilela Pereira, S. Ahuja, C. A. Bernardes, S. Dogra, T. R. Fernandez Perez Tomei, E. M. Gregores, P. G. Mercadante, C. S. Moon, S. F. Novaes, Sandra S. Padula, D. Romero Abad, J. C. Ruiz Vargas, A. Aleksandrov, R. Hadjiiska, P. Iaydjiev, M. Rodozov, S. Stoykova, G. Sultanov, M. Vutova, A. Dimitrov, I. Glushkov, L. Litov, B. Pavlov, P. Petkov, W. Fang, M. Ahmad, J. G. Bian, G. M. Chen, H. S. Chen, M. Chen, Y. Chen, T. Cheng, C. H. Jiang, D. Leggat, Z. Liu, F. Romeo, S. M. Shaheen, A. Spiezia, J. Tao, C. Wang, Z. Wang, H. Zhang, J. Zhao, Y. Ban, G. Chen, Q. Li, S. Liu, Y. Mao, S. J. Qian, D. Wang, Z. Xu, C. Avila, A. Cabrera, L. F. Chaparro Sierra, C. Florez, J. P. Gomez, C. F. González Hernández, J. D. Ruiz Alvarez, J. C. Sanabria, N. Godinovic, D. Lelas, I. Puljak, P. M. Ribeiro Cipriano, T. Sculac, Z. Antunovic, M. Kovac, V. Brigljevic, D. Ferencek, K. Kadija, S. Micanovic, L. Sudic, T. Susa, A. Attikis, G. Mavromanolakis, J. Mousa, C. Nicolaou, F. Ptochos, P. A. Razis, H. Rykaczewski, D. Tsiakkouri, M. Finger, M. Finger, E. Carrera Jarrin, E. El-khateeb, S. Elgammal, A. Mohamed, B. Calpas, M. Kadastik, M. Murumaa, L. Perrini, M. Raidal, A. Tiko, C. Veelken, P. Eerola, J. Pekkanen, M. Voutilainen, J. Härkönen, T. Järvinen, V. Karimäki, R. Kinnunen, T. Lampén, K. Lassila-Perini, S. Lehti, T. Lindén, P. Luukka, J. Tuominiemi, E. Tuovinen, L. Wendland, J. Talvitie, T. Tuuva, M. Besancon, F. Couderc, M. Dejardin, D. Denegri, B. Fabbro, J. L. Faure, C. Favaro, F. Ferri, S. Ganjour, S. Ghosh, A. Givernaud, P. Gras, G. Hamel de Monchenault, P. Jarry, I. Kucher, E. Locci, M. Machet, J. Malcles, J. Rander, A. Rosowsky, M. Titov, A. Zghiche, A. Abdulsalam, I. Antropov, S. Baffioni, F. Beaudette, P. Busson, L. Cadamuro, E. Chapon, C. Charlot, O. Davignon, R. Granier de Cassagnac, M. Jo, S. Lisniak, P. Miné, M. Nguyen, C. Ochando, G. Ortona, P. Paganini, P. Pigard, S. Regnard, R. Salerno, Y. Sirois, T. Strebler, Y. Yilmaz, A. Zabi, J.-L. Agram, J. Andrea, A. Aubin, D. Bloch, J.-M. Brom, M. Buttignol, E. C. Chabert, N. Chanon, C. Collard, E. Conte, X. Coubez, J.-C. Fontaine, D. Gelé, U. Goerlach, A.-C. Le Bihan, K. Skovpen, P. Van Hove, S. Gadrat, S. Beauceron, C. Bernet, G. Boudoul, E. Bouvier, C. A. Carrillo Montoya, R. Chierici, D. Contardo, B. Courbon, P. Depasse, H. El Mamouni, J. Fan, J. Fay, S. Gascon, M. Gouzevitch, G. Grenier, B. Ille, F. Lagarde, I. B. Laktineh, M. Lethuillier, L. Mirabito, A. L. Pequegnot, S. Perries, A. Popov, D. Sabes, V. Sordini, M. Vander Donckt, P. Verdier, S. Viret, T. Toriashvili, Z. Tsamalaidze, C. Autermann, S. Beranek, L. Feld, A. Heister, M. K. Kiesel, K. Klein, M. Lipinski, A. Ostapchuk, M. Preuten, F. Raupach, S. Schael, C. Schomakers, J. Schulz, T. Verlage, H. Weber, V. Zhukov, A. Albert, M. Brodski, E. Dietz-Laursonn, D. Duchardt, M. Endres, M. Erdmann, S. Erdweg, T. Esch, R. Fischer, A. Güth, M. Hamer, T. Hebbeker, C. Heidemann, K. Hoepfner, S. Knutzen, M. Merschmeyer, A. Meyer, P. Millet, S. Mukherjee, M. Olschewski, K. Padeken, T. Pook, M. Radziej, H. Reithler, M. Rieger, F. Scheuch, L. Sonnenschein, D. Teyssier, S. Thüer, V. Cherepanov, G. Flügge, F. Hoehle, B. Kargoll, T. Kress, A. Künsken, J. Lingemann, T. Müller, A. Nehrkorn, A. Nowack, I. M. Nugent, C. Pistone, O. Pooth, A. Stahl, M. Aldaya Martin, T. Arndt, C. Asawatangtrakuldee, K. Beernaert, O. Behnke, U. Behrens, A. A. Bin Anuar, K. Borras, A. Campbell, P. Connor, C. Contreras-Campana, F. Costanza, C. Diez Pardos, G. Dolinska, G. Eckerlin, D. Eckstein, T. Eichhorn, E. Eren, E. Gallo, J. Garay Garcia, A. Geiser, A. Gizhko, J. M. Grados Luyando, P. Gunnellini, A. Harb, J. Hauk, M. Hempel, H. Jung, A. Kalogeropoulos, O. Karacheban, M. Kasemann, J. Keaveney, C. Kleinwort, I. Korol, D. Krücker, W. Lange, A. Lelek, J. Leonard, K. Lipka, A. Lobanov, W. Lohmann, R. Mankel, I.-A. Melzer-Pellmann, A. B. Meyer, G. Mittag, J. Mnich, A. Mussgiller, E. Ntomari, D. Pitzl, R. Placakyte, A. Raspereza, B. Roland, M. Ö. Sahin, P. Saxena, T. Schoerner-Sadenius, C. Seitz, S. Spannagel, N. Stefaniuk, G. P. Van Onsem, R. Walsh, C. Wissing, V. Blobel, M. Centis Vignali, A. R. Draeger, T. Dreyer, E. Garutti, D. Gonzalez, J. Haller, M. Hoffmann, A. Junkes, R. Klanner, R. Kogler, N. Kovalchuk, T. Lapsien, T. Lenz, I. Marchesini, D. Marconi, M. Meyer, M. Niedziela, D. Nowatschin, F. Pantaleo, T. Peiffer, A. Perieanu, J. Poehlsen, C. Sander, C. Scharf, P. Schleper, A. Schmidt, S. Schumann, J. Schwandt, H. Stadie, G. Steinbrück, F. M. Stober, M. Stöver, H. Tholen, D. Troendle, E. Usai, L. Vanelderen, A. Vanhoefer, B. Vormwald, M. Akbiyik, C. Barth, S. Baur, C. Baus, J. Berger, E. Butz, R. Caspart, T. Chwalek, F. Colombo, W. De Boer, A. Dierlamm, S. Fink, B. Freund, R. Friese, M. Giffels, A. Gilbert, P. Goldenzweig, D. Haitz, F. Hartmann, S. M. Heindl, U. Husemann, I. Katkov, S. Kudella, P. Lobelle Pardo, H. Mildner, M. U. Mozer, Th. Müller, M. Plagge, G. Quast, K. Rabbertz, S. Röcker, F. Roscher, M. Schröder, I. Shvetsov, G. Sieber, H. J. Simonis, R. Ulrich, J. Wagner-Kuhr, S. Wayand, M. Weber, T. Weiler, S. Williamson, C. Wöhrmann, R. Wolf, G. Anagnostou, G. Daskalakis, T. Geralis, V. A. Giakoumopoulou, A. Kyriakis, D. Loukas, I. Topsis-Giotis, S. Kesisoglou, A. Panagiotou, N. Saoulidou, E. Tziaferi, I. Evangelou, G. Flouris, C. Foudas, P. Kokkas, N. Loukas, N. Manthos, I. Papadopoulos, E. Paradas, N. Filipovic, G. Bencze, C. Hajdu, P. Hidas, D. Horvath, F. Sikler, V. Veszpremi, G. Vesztergombi, A. J. Zsigmond, N. Beni, S. Czellar, J. Karancsi, A. Makovec, J. Molnar, Z. Szillasi, M. Bartók, P. Raics, Z. L. Trocsanyi, B. Ujvari, S. Bahinipati, S. Choudhury, P. Mal, K. Mandal, A. Nayak, D. K. Sahoo, N. Sahoo, S. K. Swain, S. Bansal, S. B. Beri, V. Bhatnagar, R. Chawla, U. Bhawandeep, A. K. Kalsi, A. Kaur, M. Kaur, R. Kumar, P. Kumari, A. Mehta, M. Mittal, J. B. Singh, G. Walia, Ashok Kumar, A. Bhardwaj, B. C. Choudhary, R. B. Garg, S. Keshri, S. Malhotra, M. Naimuddin, N. Nishu, K. Ranjan, R. Sharma, V. Sharma, R. Bhattacharya, S. Bhattacharya, K. Chatterjee, S. Dey, S. Dutt, S. Dutta, S. Ghosh, N. Majumdar, A. Modak, K. Mondal, S. Mukhopadhyay, S. Nandan, A. Purohit, A. Roy, D. Roy, S. Roy Chowdhury, S. Sarkar, M. Sharan, S. Thakur, P. K. Behera, R. Chudasama, D. Dutta, V. Jha, V. Kumar, A. K. Mohanty, P. K. Netrakanti, L. M. Pant, P. Shukla, A. Topkar, T. Aziz, S. Dugad, G. Kole, B. Mahakud, S. Mitra, G. B. Mohanty, B. Parida, N. Sur, B. Sutar, S. Banerjee, S. Bhowmik, R. K. Dewanjee, S. Ganguly, M. Guchait, Sa. Jain, S. Kumar, M. Maity, G. Majumder, K. Mazumdar, T. Sarkar, N. Wickramage, S. Chauhan, S. Dube, V. Hegde, A. Kapoor, K. Kothekar, S. Pandey, A. Rane, S. Sharma, H. Behnamian, S. Chenarani, E. Eskandari Tadavani, S. M. Etesami, A. Fahim, M. Khakzad, M. Mohammadi Najafabadi, M. Naseri, S. Paktinat Mehdiabadi, F. Rezaei Hosseinabadi, B. Safarzadeh, M. Zeinali, M. Felcini, M. Grunewald, M. Abbrescia, C. Calabria, C. Caputo, A. Colaleo, D. Creanza, L. Cristella, N. De Filippis, M. De Palma, L. Fiore, G. Iaselli, G. Maggi, M. Maggi, G. Miniello, S. My, S. Nuzzo, A. Pompili, G. Pugliese, R. Radogna, A. Ranieri, G. Selvaggi, L. Silvestris, R. Venditti, P. Verwilligen, G. Abbiendi, C. Battilana, D. Bonacorsi, S. Braibant-Giacomelli, L. Brigliadori, R. Campanini, P. Capiluppi, A. Castro, F. R. Cavallo, S. S. Chhibra, G. Codispoti, M. Cuffiani, G. M. Dallavalle, F. Fabbri, A. Fanfani, D. Fasanella, P. Giacomelli, C. Grandi, L. Guiducci, S. Marcellini, G. Masetti, A. Montanari, F. L. Navarria, A. Perrotta, A. M. Rossi, T. Rovelli, G. P. Siroli, N. Tosi, S. Albergo, M. Chiorboli, S. Costa, A. Di Mattia, F. Giordano, R. Potenza, A. Tricomi, C. Tuve, G. Barbagli, V. Ciulli, C. Civinini, R. D’Alessandro, E. Focardi, V. Gori, P. Lenzi, M. Meschini, S. Paoletti, G. Sguazzoni, L. Viliani, L. Benussi, S. Bianco, F. Fabbri, D. Piccolo, F. Primavera, V. Calvelli, F. Ferro, M. Lo Vetere, M. R. Monge, E. Robutti, S. Tosi, L. Brianza, M. E. Dinardo, S. Fiorendi, S. Gennai, A. Ghezzi, P. Govoni, M. Malberti, S. Malvezzi, R. A. Manzoni, D. Menasce, L. Moroni, M. Paganoni, D. Pedrini, S. Pigazzini, S. Ragazzi, T. Tabarelli de Fatis, S. Buontempo, N. Cavallo, G. De Nardo, S. Di Guida, M. Esposito, F. Fabozzi, F. Fienga, A. O. M. Iorio, G. Lanza, L. Lista, S. Meola, P. Paolucci, C. Sciacca, F. Thyssen, P. Azzi, N. Bacchetta, L. Benato, D. Bisello, A. Boletti, R. Carlin, A. Carvalho Antunes De Oliveira, P. Checchia, M. Dall’Osso, P. De Castro Manzano, T. Dorigo, U. Dosselli, F. Gasparini, U. Gasparini, A. Gozzelino, S. Lacaprara, M. Margoni, A. T. Meneguzzo, J. Pazzini, N. Pozzobon, P. Ronchese, F. Simonetto, E. Torassa, M. Zanetti, P. Zotto, G. Zumerle, A. Braghieri, A. Magnani, P. Montagna, S. P. Ratti, V. Re, C. Riccardi, P. Salvini, I. Vai, P. Vitulo, L. Alunni Solestizi, G. M. Bilei, D. Ciangottini, L. Fanò, P. Lariccia, R. Leonardi, G. Mantovani, M. Menichelli, A. Saha, A. Santocchia, K. Androsov, P. Azzurri, G. Bagliesi, J. Bernardini, T. Boccali, R. Castaldi, M. A. Ciocci, R. Dell’Orso, S. Donato, G. Fedi, A. Giassi, M. T. Grippo, F. Ligabue, T. Lomtadze, L. Martini, A. Messineo, F. Palla, A. Rizzi, A. Savoy-Navarro, P. Spagnolo, R. Tenchini, G. Tonelli, A. Venturi, P. G. Verdini, L. Barone, F. Cavallari, M. Cipriani, D. Del Re, M. Diemoz, S. Gelli, E. Longo, F. Margaroli, B. Marzocchi, P. Meridiani, G. Organtini, R. Paramatti, F. Preiato, S. Rahatlou, C. Rovelli, F. Santanastasio, N. Amapane, R. Arcidiacono, S. Argiro, M. Arneodo, N. Bartosik, R. Bellan, C. Biino, N. Cartiglia, F. Cenna, M. Costa, R. Covarelli, A. Degano, N. Demaria, L. Finco, B. Kiani, C. Mariotti, S. Maselli, E. Migliore, V. Monaco, E. Monteil, M. M. Obertino, L. Pacher, N. Pastrone, M. Pelliccioni, G. L. Pinna Angioni, F. Ravera, A. Romero, M. Ruspa, R. Sacchi, K. Shchelina, V. Sola, A. Solano, A. Staiano, P. Traczyk, S. Belforte, M. Casarsa, F. Cossutti, G. Della Ricca, A. Zanetti, D. H. Kim, G. N. Kim, M. S. Kim, S. Lee, S. W. Lee, Y. D. Oh, S. Sekmen, D. C. Son, Y. C. Yang, A. Lee, H. Kim, J. A. Brochero Cifuentes, T. J. Kim, S. Cho, S. Choi, Y. Go, D. Gyun, S. Ha, B. Hong, Y. Jo, Y. Kim, B. Lee, K. Lee, K. S. Lee, S. Lee, J. Lim, S. K. Park, Y. Roh, J. Almond, J. Kim, H. Lee, S. B. Oh, B. C. Radburn-Smith, S. H. Seo, U. K. Yang, H. D. Yoo, G. B. Yu, M. Choi, H. Kim, J. H. Kim, J. S. H. Lee, I. C. Park, G. Ryu, M. S. Ryu, Y. Choi, J. Goh, C. Hwang, J. Lee, I. Yu, V. Dudenas, A. Juodagalvis, J. Vaitkus, I. Ahmed, Z. A. Ibrahim, J. R. Komaragiri, M. A. B. Md Ali, F. Mohamad Idris, W. A. T. Wan Abdullah, M. N. Yusli, Z. Zolkapli, H. Castilla-Valdez, E. De La Cruz-Burelo, I. Heredia-De La Cruz, A. Hernandez-Almada, R. Lopez-Fernandez, R. Magaña Villalba, J. Mejia Guisao, A. Sanchez-Hernandez, S. Carrillo Moreno, C. Oropeza Barrera, F. Vazquez Valencia, S. Carpinteyro, I. Pedraza, H. A. Salazar Ibarguen, C. Uribe Estrada, A. Morelos Pineda, D. Krofcheck, P. H. Butler, A. Ahmad, M. Ahmad, Q. Hassan, H. R. Hoorani, W. A. Khan, A. Saddique, M. A. Shah, M. Shoaib, M. Waqas, H. Bialkowska, M. Bluj, B. Boimska, T. Frueboes, M. Górski, M. Kazana, K. Nawrocki, K. Romanowska-Rybinska, M. Szleper, P. Zalewski, K. Bunkowski, A. Byszuk, K. Doroba, A. Kalinowski, M. Konecki, J. Krolikowski, M. Misiura, M. Olszewski, M. Walczak, P. Bargassa, C. Beirão Da Cruz E Silva, A. Di Francesco, P. Faccioli, P. G. Ferreira Parracho, M. Gallinaro, J. Hollar, N. Leonardo, L. Lloret Iglesias, M. V. Nemallapudi, J. Rodrigues Antunes, J. Seixas, O. Toldaiev, D. Vadruccio, J. Varela, P. Vischia, S. Afanasiev, P. Bunin, M. Gavrilenko, I. Golutvin, I. Gorbunov, A. Kamenev, V. Karjavin, A. Lanev, A. Malakhov, V. Matveev, V. Palichik, V. Perelygin, S. Shmatov, S. Shulha, N. Skatchkov, V. Smirnov, N. Voytishin, A. Zarubin, L. Chtchipounov, V. Golovtsov, Y. Ivanov, V. Kim, E. Kuznetsova, V. Murzin, V. Oreshkin, V. Sulimov, A. Vorobyev, Yu. Andreev, A. Dermenev, S. Gninenko, N. Golubev, A. Karneyeu, M. Kirsanov, N. Krasnikov, A. Pashenkov, D. Tlisov, A. Toropin, V. Epshteyn, V. Gavrilov, N. Lychkovskaya, V. Popov, I. Pozdnyakov, G. Safronov, A. Spiridonov, M. Toms, E. Vlasov, A. Zhokin, A. Bylinkin, M. Chadeeva, V. Rusinov, E. Tarkovskii, V. Andreev, M. Azarkin, I. Dremin, M. Kirakosyan, A. Leonidov, S. V. Rusakov, A. Terkulov, A. Baskakov, A. Belyaev, E. Boos, M. Dubinin, L. Dudko, A. Ershov, A. Gribushin, V. Klyukhin, O. Kodolova, I. Lokhtin, I. Miagkov, S. Obraztsov, S. Petrushanko, V. Savrin, A. Snigirev, V. Blinov, Y. Skovpen, D. Shtol, I. Azhgirey, I. Bayshev, S. Bitioukov, D. Elumakhov, V. Kachanov, A. Kalinin, D. Konstantinov, V. Krychkine, V. Petrov, R. Ryutin, A. Sobol, S. Troshin, N. Tyurin, A. Uzunian, A. Volkov, P. Adzic, P. Cirkovic, D. Devetak, M. Dordevic, J. Milosevic, V. Rekovic, J. Alcaraz Maestre, M. Barrio Luna, E. Calvo, M. Cerrada, M. Chamizo Llatas, N. Colino, B. De La Cruz, A. Delgado Peris, A. Escalante Del Valle, C. Fernandez Bedoya, J. P. Fernández Ramos, J. Flix, M. C. Fouz, P. Garcia-Abia, O. Gonzalez Lopez, S. Goy Lopez, J. M. Hernandez, M. I. Josa, E. Navarro De Martino, A. Pérez-Calero Yzquierdo, J. Puerta Pelayo, A. Quintario Olmeda, I. Redondo, L. Romero, M. S. Soares, J. F. de Trocóniz, M. Missiroli, D. Moran, J. Cuevas, J. Fernandez Menendez, I. Gonzalez Caballero, J. R. González Fernández, E. Palencia Cortezon, S. Sanchez Cruz, I. Suárez Andrés, J. M. Vizan Garcia, I. J. Cabrillo, A. Calderon, J. R. Castiñeiras De Saa, E. Curras, M. Fernandez, J. Garcia-Ferrero, G. Gomez, A. Lopez Virto, J. Marco, C. Martinez Rivero, F. Matorras, J. Piedra Gomez, T. Rodrigo, A. Ruiz-Jimeno, L. Scodellaro, N. Trevisani, I. Vila, R. Vilar Cortabitarte, D. Abbaneo, E. Auffray, G. Auzinger, M. Bachtis, P. Baillon, A. H. Ball, D. Barney, P. Bloch, A. Bocci, A. Bonato, C. Botta, T. Camporesi, R. Castello, M. Cepeda, G. Cerminara, M. D’Alfonso, D. d’Enterria, A. Dabrowski, V. Daponte, A. David, M. De Gruttola, A. De Roeck, E. Di Marco, M. Dobson, B. Dorney, T. du Pree, D. Duggan, M. Dünser, N. Dupont, A. Elliott-Peisert, S. Fartoukh, G. Franzoni, J. Fulcher, W. Funk, D. Gigi, K. Gill, M. Girone, F. Glege, D. Gulhan, S. Gundacker, M. Guthoff, J. Hammer, P. Harris, J. Hegeman, V. Innocente, P. Janot, J. Kieseler, H. Kirschenmann, V. Knünz, A. Kornmayer, M. J. Kortelainen, K. Kousouris, M. Krammer, C. Lange, P. Lecoq, C. Lourenço, M. T. Lucchini, L. Malgeri, M. Mannelli, A. Martelli, F. Meijers, J. A. Merlin, S. Mersi, E. Meschi, P. Milenovic, F. Moortgat, S. Morovic, M. Mulders, H. Neugebauer, S. Orfanelli, L. Orsini, L. Pape, E. Perez, M. Peruzzi, A. Petrilli, G. Petrucciani, A. Pfeiffer, M. Pierini, A. Racz, T. Reis, G. Rolandi, M. Rovere, M. Ruan, H. Sakulin, J. B. Sauvan, C. Schäfer, C. Schwick, M. Seidel, A. Sharma, P. Silva, P. Sphicas, J. Steggemann, M. Stoye, Y. Takahashi, M. Tosi, D. Treille, A. Triossi, A. Tsirou, V. Veckalns, G. I. Veres, M. Verweij, N. Wardle, H. K. Wöhri, A. Zagozdzinska, W. D. Zeuner, W. Bertl, K. Deiters, W. Erdmann, R. Horisberger, Q. Ingram, H. C. Kaestli, D. Kotlinski, U. Langenegger, T. Rohe, F. Bachmair, L. Bäni, L. Bianchini, B. Casal, G. Dissertori, M. Dittmar, M. Donegà, C. Grab, C. Heidegger, D. Hits, J. Hoss, G. Kasieczka, P. Lecomte, W. Lustermann, B. Mangano, M. Marionneau, P. Martinez Ruiz del Arbol, M. Masciovecchio, M. T. Meinhard, D. Meister, F. Micheli, P. Musella, F. Nessi-Tedaldi, F. Pandolfi, J. Pata, F. Pauss, G. Perrin, L. Perrozzi, M. Quittnat, M. Rossini, M. Schönenberger, A. Starodumov, V. R. Tavolaro, K. Theofilatos, R. Wallny, T. K. Aarrestad, C. Amsler, L. Caminada, M. F. Canelli, A. De Cosa, C. Galloni, A. Hinzmann, T. Hreus, B. Kilminster, J. Ngadiuba, D. Pinna, G. Rauco, P. Robmann, D. Salerno, Y. Yang, A. Zucchetta, V. Candelise, T. H. Doan, Sh. Jain, R. Khurana, M. Konyushikhin, C. M. Kuo, W. Lin, Y. J. Lu, A. Pozdnyakov, S. S. Yu, Arun Kumar, P. Chang, Y. H. Chang, Y. W. Chang, Y. Chao, K. F. Chen, P. H. Chen, C. Dietz, F. Fiori, W.-S. Hou, Y. Hsiung, Y. F. Liu, R.-S. Lu, M. Miñano Moya, E. Paganis, A. Psallidas, J. F. Tsai, Y. M. Tzeng, B. Asavapibhop, G. Singh, N. Srimanobhas, N. Suwonjandee, A. Adiguzel, S. Cerci, S. Damarseckin, Z. S. Demiroglu, C. Dozen, I. Dumanoglu, S. Girgis, G. Gokbulut, Y. Guler, I. Hos, E. E. Kangal, O. Kara, A. Kayis Topaksu, U. Kiminsu, M. Oglakci, G. Onengut, K. Ozdemir, D. Sunar Cerci, H. Topakli, S. Turkcapar, I. S. Zorbakir, C. Zorbilmez, B. Bilin, S. Bilmis, B. Isildak, G. Karapinar, M. Yalvac, M. Zeyrek, E. Gülmez, M. Kaya, O. Kaya, E. A. Yetkin, T. Yetkin, A. Cakir, K. Cankocak, S. Sen, B. Grynyov, L. Levchuk, P. Sorokin, R. Aggleton, F. Ball, L. Beck, J. J. Brooke, D. Burns, E. Clement, D. Cussans, H. Flacher, J. Goldstein, M. Grimes, G. P. Heath, H. F. Heath, J. Jacob, L. Kreczko, C. Lucas, D. M. Newbold, S. Paramesvaran, A. Poll, T. Sakuma, S. Seif El Nasr-storey, D. Smith, V. J. Smith, K. W. Bell, A. Belyaev, C. Brew, R. M. Brown, L. Calligaris, D. Cieri, D. J. A. Cockerill, J. A. Coughlan, K. Harder, S. Harper, E. Olaiya, D. Petyt, C. H. Shepherd-Themistocleous, A. Thea, I. R. Tomalin, T. Williams, M. Baber, R. Bainbridge, O. Buchmuller, A. Bundock, D. Burton, S. Casasso, M. Citron, D. Colling, L. Corpe, P. Dauncey, G. Davies, A. De Wit, M. Della Negra, R. Di Maria, P. Dunne, A. Elwood, D. Futyan, Y. Haddad, G. Hall, G. Iles, T. James, R. Lane, C. Laner, R. Lucas, L. Lyons, A.-M. Magnan, S. Malik, L. Mastrolorenzo, J. Nash, A. Nikitenko, J. Pela, B. Penning, M. Pesaresi, D. M. Raymond, A. Richards, A. Rose, C. Seez, S. Summers, A. Tapper, K. Uchida, M. Vazquez Acosta, T. Virdee, J. Wright, S. C. Zenz, J. E. Cole, P. R. Hobson, A. Khan, P. Kyberd, D. Leslie, I. D. Reid, P. Symonds, L. Teodorescu, M. Turner, A. Borzou, K. Call, J. Dittmann, K. Hatakeyama, H. Liu, N. Pastika, O. Charaf, S. I. Cooper, C. Henderson, P. Rumerio, C. West, D. Arcaro, A. Avetisyan, T. Bose, D. Gastler, D. Rankin, C. Richardson, J. Rohlf, L. Sulak, D. Zou, G. Benelli, E. Berry, D. Cutts, A. Garabedian, J. Hakala, U. Heintz, J. M. Hogan, O. Jesus, K. H. M. Kwok, E. Laird, G. Landsberg, Z. Mao, M. Narain, S. Piperov, S. Sagir, E. Spencer, R. Syarif, R. Breedon, G. Breto, D. Burns, M. Calderon De La Barca Sanchez, S. Chauhan, M. Chertok, J. Conway, R. Conway, P. T. Cox, R. Erbacher, C. Flores, G. Funk, M. Gardner, W. Ko, R. Lander, C. Mclean, M. Mulhearn, D. Pellett, J. Pilot, S. Shalhout, J. Smith, M. Squires, D. Stolp, M. Tripathi, S. Wilbur, R. Yohay, C. Bravo, R. Cousins, A. Dasgupta, P. Everaerts, A. Florent, J. Hauser, M. Ignatenko, N. Mccoll, D. Saltzberg, C. Schnaible, E. Takasugi, V. Valuev, M. Weber, K. Burt, R. Clare, J. Ellison, J. W. Gary, S. M. A. Ghiasi Shirazi, G. Hanson, J. Heilman, P. Jandir, E. Kennedy, F. Lacroix, O. R. Long, M. Olmedo Negrete, M. I. Paneva, A. Shrinivas, W. Si, H. Wei, S. Wimpenny, B. R. Yates, J. G. Branson, G. B. Cerati, S. Cittolin, M. Derdzinski, R. Gerosa, A. Holzner, D. Klein, V. Krutelyov, J. Letts, I. Macneill, D. Olivito, S. Padhi, M. Pieri, M. Sani, V. Sharma, S. Simon, M. Tadel, A. Vartak, S. Wasserbaech, C. Welke, J. Wood, F. Würthwein, A. Yagil, G. Zevi Della Porta, N. Amin, R. Bhandari, J. Bradmiller-Feld, C. Campagnari, A. Dishaw, V. Dutta, K. Flowers, M. Franco Sevilla, P. Geffert, C. George, F. Golf, L. Gouskos, J. Gran, R. Heller, J. Incandela, S. D. Mullin, A. Ovcharova, H. Qu, J. Richman, D. Stuart, I. Suarez, J. Yoo, D. Anderson, A. Apresyan, J. Bendavid, A. Bornheim, J. Bunn, Y. Chen, J. Duarte, J. M. Lawhorn, A. Mott, H. B. Newman, C. Pena, M. Spiropulu, J. R. Vlimant, S. Xie, R. Y. Zhu, M. B. Andrews, V. Azzolini, T. Ferguson, M. Paulini, J. Russ, M. Sun, H. Vogel, I. Vorobiev, M. Weinberg, J. P. Cumalat, W. T. Ford, F. Jensen, A. Johnson, M. Krohn, T. Mulholland, K. Stenson, S. R. Wagner, J. Alexander, J. Chaves, J. Chu, S. Dittmer, K. Mcdermott, N. Mirman, G. Nicolas Kaufman, J. R. Patterson, A. Rinkevicius, A. Ryd, L. Skinnari, L. Soffi, S. M. Tan, Z. Tao, J. Thom, J. Tucker, P. Wittich, M. Zientek, D. Winn, S. Abdullin, M. Albrow, G. Apollinari, S. Banerjee, L. A. T. Bauerdick, A. Beretvas, J. Berryhill, P. C. Bhat, G. Bolla, K. Burkett, J. N. Butler, H. W. K. Cheung, F. Chlebana, S. Cihangir, M. Cremonesi, V. D. Elvira, I. Fisk, J. Freeman, E. Gottschalk, L. Gray, D. Green, S. Grünendahl, O. Gutsche, D. Hare, R. M. Harris, S. Hasegawa, J. Hirschauer, Z. Hu, B. Jayatilaka, S. Jindariani, M. Johnson, U. Joshi, B. Klima, B. Kreis, S. Lammel, J. Linacre, D. Lincoln, R. Lipton, M. Liu, T. Liu, R. Lopes De Sá, J. Lykken, K. Maeshima, N. Magini, J. M. Marraffino, S. Maruyama, D. Mason, P. McBride, P. Merkel, S. Mrenna, S. Nahn, C. Newman-Holmes, V. O’Dell, K. Pedro, O. Prokofyev, G. Rakness, L. Ristori, E. Sexton-Kennedy, A. Soha, W. J. Spalding, L. Spiegel, S. Stoynev, J. Strait, N. Strobbe, L. Taylor, S. Tkaczyk, N. V. Tran, L. Uplegger, E. W. Vaandering, C. Vernieri, M. Verzocchi, R. Vidal, M. Wang, H. A. Weber, A. Whitbeck, Y. Wu, D. Acosta, P. Avery, P. Bortignon, D. Bourilkov, A. Brinkerhoff, A. Carnes, M. Carver, D. Curry, S. Das, R. D. Field, I. K. Furic, J. Konigsberg, A. Korytov, J. F. Low, P. Ma, K. Matchev, H. Mei, G. Mitselmakher, D. Rank, L. Shchutska, D. Sperka, L. Thomas, J. Wang, S. Wang, J. Yelton, S. Linn, P. Markowitz, G. Martinez, J. L. Rodriguez, A. Ackert, J. R. Adams, T. Adams, A. Askew, S. Bein, B. Diamond, S. Hagopian, V. Hagopian, K. F. Johnson, A. Khatiwada, H. Prosper, A. Santra, M. M. Baarmand, V. Bhopatkar, S. Colafranceschi, M. Hohlmann, D. Noonan, T. Roy, F. Yumiceva, M. R. Adams, L. Apanasevich, D. Berry, R. R. Betts, I. Bucinskaite, R. Cavanaugh, O. Evdokimov, L. Gauthier, C. E. Gerber, D. J. Hofman, K. Jung, P. Kurt, C. O’Brien, I. D. Sandoval Gonzalez, P. Turner, N. Varelas, H. Wang, Z. Wu, M. Zakaria, J. Zhang, B. Bilki, W. Clarida, K. Dilsiz, S. Durgut, R. P. Gandrajula, M. Haytmyradov, V. Khristenko, J.-P. Merlo, H. Mermerkaya, A. Mestvirishvili, A. Moeller, J. Nachtman, H. Ogul, Y. Onel, F. Ozok, A. Penzo, C. Snyder, E. Tiras, J. Wetzel, K. Yi, I. Anderson, B. Blumenfeld, A. Cocoros, N. Eminizer, D. Fehling, L. Feng, A. V. Gritsan, P. Maksimovic, C. Martin, M. Osherson, J. Roskes, U. Sarica, M. Swartz, M. Xiao, Y. Xin, C. You, A. Al-bataineh, P. Baringer, A. Bean, S. Boren, J. Bowen, C. Bruner, J. Castle, L. Forthomme, R. P. Kenny, S. Khalil, A. Kropivnitskaya, D. Majumder, W. Mcbrayer, M. Murray, S. Sanders, R. Stringer, J. D. Tapia Takaki, Q. Wang, A. Ivanov, K. Kaadze, Y. Maravin, A. Mohammadi, L. K. Saini, N. Skhirtladze, S. Toda, F. Rebassoo, D. Wright, C. Anelli, A. Baden, O. Baron, A. Belloni, B. Calvert, S. C. Eno, C. Ferraioli, J. A. Gomez, N. J. Hadley, S. Jabeen, R. G. Kellogg, T. Kolberg, J. Kunkle, Y. Lu, A. C. Mignerey, F. Ricci-Tam, Y. H. Shin, A. Skuja, M. B. Tonjes, S. C. Tonwar, D. Abercrombie, B. Allen, A. Apyan, R. Barbieri, A. Baty, R. Bi, K. Bierwagen, S. Brandt, W. Busza, I. A. Cali, Z. Demiragli, L. Di Matteo, G. Gomez Ceballos, M. Goncharov, D. Hsu, Y. Iiyama, G. M. Innocenti, M. Klute, D. Kovalskyi, K. Krajczar, Y. S. Lai, Y.-J. Lee, A. Levin, P. D. Luckey, B. Maier, A. C. Marini, C. Mcginn, C. Mironov, S. Narayanan, X. Niu, C. Paus, C. Roland, G. Roland, J. Salfeld-Nebgen, G. S. F. Stephans, K. Sumorok, K. Tatar, M. Varma, D. Velicanu, J. Veverka, J. Wang, T. W. Wang, B. Wyslouch, M. Yang, V. Zhukova, A. C. Benvenuti, R. M. Chatterjee, A. Evans, A. Finkel, A. Gude, P. Hansen, S. Kalafut, S. C. Kao, Y. Kubota, Z. Lesko, J. Mans, S. Nourbakhsh, N. Ruckstuhl, R. Rusack, N. Tambe, J. Turkewitz, J. G. Acosta, S. Oliveros, E. Avdeeva, R. Bartek, K. Bloom, D. R. Claes, A. Dominguez, C. Fangmeier, R. Gonzalez Suarez, R. Kamalieddin, I. Kravchenko, A. Malta Rodrigues, F. Meier, J. Monroy, J. E. Siado, G. R. Snow, B. Stieger, M. Alyari, J. Dolen, J. George, A. Godshalk, C. Harrington, I. Iashvili, J. Kaisen, A. Kharchilava, A. Kumar, A. Parker, S. Rappoccio, B. Roozbahani, G. Alverson, E. Barberis, A. Hortiangtham, A. Massironi, D. M. Morse, D. Nash, T. Orimoto, R. Teixeira De Lima, D. Trocino, R.-J. Wang, D. Wood, S. Bhattacharya, K. A. Hahn, A. Kubik, A. Kumar, N. Mucia, N. Odell, B. Pollack, M. H. Schmitt, K. Sung, M. Trovato, M. Velasco, N. Dev, M. Hildreth, K. Hurtado Anampa, C. Jessop, D. J. Karmgard, N. Kellams, K. Lannon, N. Marinelli, F. Meng, C. Mueller, Y. Musienko, M. Planer, A. Reinsvold, R. Ruchti, G. Smith, S. Taroni, M. Wayne, M. Wolf, A. Woodard, J. Alimena, L. Antonelli, J. Brinson, B. Bylsma, L. S. Durkin, S. Flowers, B. Francis, A. Hart, C. Hill, R. Hughes, W. Ji, B. Liu, W. Luo, D. Puigh, B. L. Winer, H. W. Wulsin, S. Cooperstein, O. Driga, P. Elmer, J. Hardenbrook, P. Hebda, D. Lange, J. Luo, D. Marlow, J. Mc Donald, T. Medvedeva, K. Mei, M. Mooney, J. Olsen, C. Palmer, P. Piroué, D. Stickland, A. Svyatkovskiy, C. Tully, A. Zuranski, S. Malik, A. Barker, V. E. Barnes, S. Folgueras, L. Gutay, M. K. Jha, M. Jones, A. W. Jung, D. H. Miller, N. Neumeister, J. F. Schulte, X. Shi, J. Sun, F. Wang, W. Xie, L. Xu, N. Parashar, J. Stupak, A. Adair, B. Akgun, Z. Chen, K. M. Ecklund, F. J. M. Geurts, M. Guilbaud, W. Li, B. Michlin, M. Northup, B. P. Padley, R. Redjimi, J. Roberts, J. Rorie, Z. Tu, J. Zabel, B. Betchart, A. Bodek, P. de Barbaro, R. Demina, Y. T. Duh, T. Ferbel, M. Galanti, A. Garcia-Bellido, J. Han, O. Hindrichs, A. Khukhunaishvili, K. H. Lo, P. Tan, M. Verzetti, A. Agapitos, J. P. Chou, E. Contreras-Campana, Y. Gershtein, T. A. Gómez Espinosa, E. Halkiadakis, M. Heindl, D. Hidas, E. Hughes, S. Kaplan, R. Kunnawalkam Elayavalli, S. Kyriacou, A. Lath, K. Nash, H. Saka, S. Salur, S. Schnetzer, D. Sheffield, S. Somalwar, R. Stone, S. Thomas, P. Thomassen, M. Walker, A. G. Delannoy, M. Foerster, J. Heideman, G. Riley, K. Rose, S. Spanier, K. Thapa, O. Bouhali, A. Celik, M. Dalchenko, M. De Mattia, A. Delgado, S. Dildick, R. Eusebi, J. Gilmore, T. Huang, E. Juska, T. Kamon, R. Mueller, Y. Pakhotin, R. Patel, A. Perloff, L. Perniè, D. Rathjens, A. Rose, A. Safonov, A. Tatarinov, K. A. Ulmer, N. Akchurin, C. Cowden, J. Damgov, F. De Guio, C. Dragoiu, P. R. Dudero, J. Faulkner, E. Gurpinar, S. Kunori, K. Lamichhane, S. W. Lee, T. Libeiro, T. Peltola, S. Undleeb, I. Volobouev, Z. Wang, S. Greene, A. Gurrola, R. Janjam, W. Johns, C. Maguire, A. Melo, H. Ni, P. Sheldon, S. Tuo, J. Velkovska, Q. Xu, M. W. Arenton, P. Barria, B. Cox, J. Goodell, R. Hirosky, A. Ledovskoy, H. Li, C. Neu, T. Sinthuprasith, X. Sun, Y. Wang, E. Wolfe, F. Xia, C. Clarke, R. Harr, P. E. Karchin, J. Sturdy, D. A. Belknap, C. Caillol, S. Dasu, L. Dodd, S. Duric, B. Gomber, M. Grothe, M. Herndon, A. Hervé, P. Klabbers, A. Lanaro, A. Levine, K. Long, R. Loveless, I. Ojalvo, T. Perry, G. A. Pierro, G. Polese, T. Ruggles, A. Savin, N. Smith, W. H. Smith, D. Taylor, N. Woods, The CMS Collaboration

**Affiliations:** 1 Yerevan Physics Institute, Yerevan, Armenia; 2 Institut für Hochenergiephysik, Vienna, Austria; 3 Institute for Nuclear Problems, Minsk, Belarus; 4 National Centre for Particle and High Energy Physics, Minsk, Belarus; 5 Universiteit Antwerpen, Antwerpen, Belgium; 6 Vrije Universiteit Brussel, Brussel, Belgium; 7 Université Libre de Bruxelles, Bruxelles, Belgium; 8 Ghent University, Ghent, Belgium; 9 Université Catholique de Louvain, Louvain-la-Neuve, Belgium; 10 Université de Mons, Mons, Belgium; 11 Centro Brasileiro de Pesquisas Fisicas, Rio de Janeiro, Brazil; 12 Universidade do Estado do Rio de Janeiro, Rio de Janeiro, Brazil; 13 Universidade Estadual Paulista, Universidade Federal do ABC, São Paulo, Brazil; 14 Institute for Nuclear Research and Nuclear Energy, Sofia, Bulgaria; 15 University of Sofia, Sofia, Bulgaria; 16 Beihang University, Beijing, China; 17 Institute of High Energy Physics, Beijing, China; 18 State Key Laboratory of Nuclear Physics and Technology, Peking University, Beijing, China; 19 Universidad de Los Andes, Bogota, Colombia; 20 Faculty of Electrical Engineering, Mechanical Engineering and Naval Architecture, University of Split, Split, Croatia; 21 University of Split, Faculty of Science, Split, Croatia; 22 Institute Rudjer Boskovic, Zagreb, Croatia; 23 University of Cyprus, Nicosia, Cyprus; 24 Charles University, Prague, Czech Republic; 25 Universidad San Francisco de Quito, Quito, Ecuador; 26 Academy of Scientific Research and Technology of the Arab Republic of Egypt, Egyptian Network of High Energy Physics, Cairo, Egypt; 27 National Institute of Chemical Physics and Biophysics, Tallinn, Estonia; 28 Department of Physics, University of Helsinki, Helsinki, Finland; 29 Helsinki Institute of Physics, Helsinki, Finland; 30 Lappeenranta University of Technology, Lappeenranta, Finland; 31 IRFU, CEA, Université Paris-Saclay, Gif-sur-Yvette, France; 32 Laboratoire Leprince-Ringuet, Ecole Polytechnique, IN2P3-CNRS, Palaiseau, France; 33 Institut Pluridisciplinaire Hubert Curien, Université de Strasbourg, Université de Haute Alsace Mulhouse, CNRS/IN2P3, Strasbourg, France; 34 Centre de Calcul de l’Institut National de Physique Nucleaire et de Physique des Particules, CNRS/IN2P3, Villeurbanne, France; 35 Université de Lyon, Université Claude Bernard Lyon 1, CNRS-IN2P3, Institut de Physique Nucléaire de Lyon, Villeurbanne, France; 36 Georgian Technical University, Tbilisi, Georgia; 37 Tbilisi State University, Tbilisi, Georgia; 38 RWTH Aachen University, I. Physikalisches Institut, Aachen, Germany; 39 RWTH Aachen University, III. Physikalisches Institut A, Aachen, Germany; 40 RWTH Aachen University, III. Physikalisches Institut B, Aachen, Germany; 41 Deutsches Elektronen-Synchrotron, Hamburg, Germany; 42 University of Hamburg, Hamburg, Germany; 43 Institut für Experimentelle Kernphysik, Karlsruhe, Germany; 44 Institute of Nuclear and Particle Physics (INPP), NCSR Demokritos, Aghia Paraskevi, Greece; 45 National and Kapodistrian University of Athens, Athens, Greece; 46 University of Ioánnina, Ioánnina, Greece; 47 MTA-ELTE Lendület CMS Particle and Nuclear Physics Group, Eötvös Loránd University, Budapest, Hungary; 48 Wigner Research Centre for Physics, Budapest, Hungary; 49 Institute of Nuclear Research ATOMKI, Debrecen, Hungary; 50 University of Debrecen, Debrecen, Hungary; 51 National Institute of Science Education and Research, Bhubaneswar, India; 52 Panjab University, Chandigarh, India; 53 University of Delhi, Delhi, India; 54 Saha Institute of Nuclear Physics, Kolkata, India; 55 Indian Institute of Technology Madras, Madras, India; 56 Bhabha Atomic Research Centre, Mumbai, India; 57 Tata Institute of Fundamental Research-A, Mumbai, India; 58 Tata Institute of Fundamental Research-B, Mumbai, India; 59 Indian Institute of Science Education and Research (IISER), Pune, India; 60 Institute for Research in Fundamental Sciences (IPM), Tehran, Iran; 61 University College Dublin, Dublin, Ireland; 62 INFN Sezione di Bari, Università di Bari, Politecnico di Bari, Bari, Italy; 63 INFN Sezione di Bologna, Università di Bologna, Bologna, Italy; 64 INFN Sezione di Catania, Università di Catania, Catania, Italy; 65 INFN Sezione di Firenze, Università di Firenze, Florence, Italy; 66 INFN Laboratori Nazionali di Frascati, Frascati, Italy; 67 INFN Sezione di Genova, Università di Genova, Genoa, Italy; 68 INFN Sezione di Milano-Bicocca, Università di Milano-Bicocca, Milan, Italy; 69 INFN Sezione di Napoli, Università di Napoli ’Federico II’, Napoli, Italy, Università della Basilicata, Potenza, Italy, Università G. Marconi, Rome, Italy; 70 INFN Sezione di Padova, Università di Padova Padova Italy, Università di Trento, Trento, Italy; 71 INFN Sezione di Pavia, Università di Pavia, Pavia, Italy; 72 INFN Sezione di Perugia, Università di Perugia, Perugia, Italy; 73 INFN Sezione di Pisa, Università di Pisa, Scuola Normale Superiore di Pisa, Pisa, Italy; 74 INFN Sezione di Roma, Università di Roma, Rome, Italy; 75 INFN Sezione di Torino, Università di Torino, Turin, Italy, Università del Piemonte Orientale, Novara, Italy; 76 INFN Sezione di Trieste, Università di Trieste, Trieste, Italy; 77 Kyungpook National University, Taegu, Korea; 78 Chonbuk National University, Jeonju, Korea; 79 Institute for Universe and Elementary Particles, Chonnam National University, Kwangju, Korea; 80 Hanyang University, Seoul, Korea; 81 Korea University, Seoul, Korea; 82 Seoul National University, Seoul, Korea; 83 University of Seoul, Seoul, Korea; 84 Sungkyunkwan University, Suwon, Korea; 85 Vilnius University, Vilnius, Lithuania; 86 National Centre for Particle Physics, Universiti Malaya, Kuala Lumpur, Malaysia; 87 Centro de Investigacion y de Estudios Avanzados del IPN, Mexico City, Mexico; 88 Universidad Iberoamericana, Mexico City, Mexico; 89 Benemerita Universidad Autonoma de Puebla, Puebla, Mexico; 90 Universidad Autónoma de San Luis Potosí, San Luis Potosí, Mexico; 91 University of Auckland, Auckland, New Zealand; 92 University of Canterbury, Christchurch, New Zealand; 93 National Centre for Physics, Quaid-I-Azam University, Islamabad, Pakistan; 94 National Centre for Nuclear Research, Swierk, Poland; 95 Institute of Experimental Physics, Faculty of Physics, University of Warsaw, Warsaw, Poland; 96 Laboratório de Instrumentação e Física Experimental de Partículas, Lisbon, Portugal; 97 Joint Institute for Nuclear Research, Dubna, Russia; 98 Petersburg Nuclear Physics Institute, Gatchina (St. Petersburg), Russia; 99 Institute for Nuclear Research, Moscow, Russia; 100 Institute for Theoretical and Experimental Physics, Moscow, Russia; 101 Moscow Institute of Physics and Technology, Moscow, Russia; 102 National Research Nuclear University ‘Moscow Engineering Physics Institute’ (MEPhI), Moscow, Russia; 103 P.N. Lebedev Physical Institute, Moscow, Russia; 104 Skobeltsyn Institute of Nuclear Physics, Lomonosov Moscow State University, Moscow, Russia; 105 Novosibirsk State University (NSU), Novosibirsk, Russia; 106 State Research Center of Russian Federation, Institute for High Energy Physics, Protvino, Russia; 107 Faculty of Physics and Vinca Institute of Nuclear Sciences, University of Belgrade, Belgrade, Serbia; 108 Centro de Investigaciones Energéticas Medioambientales y Tecnológicas (CIEMAT), Madrid, Spain; 109 Universidad Autónoma de Madrid, Madrid, Spain; 110 Universidad de Oviedo, Oviedo, Spain; 111 Instituto de Física de Cantabria (IFCA), CSIC-Universidad de Cantabria, Santander, Spain; 112 CERN, European Organization for Nuclear Research, Geneva, Switzerland; 113 Paul Scherrer Institut, Villigen, Switzerland; 114 Institute for Particle Physics, ETH Zurich, Zurich, Switzerland; 115 Universität Zürich, Zurich, Switzerland; 116 National Central University, Chung-Li, Taiwan; 117 National Taiwan University (NTU), Taipei, Taiwan; 118 Department of Physics, Faculty of Science, Chulalongkorn University, Bangkok, Thailand; 119 Cukurova University, Adana, Turkey; 120 Physics Department, Middle East Technical University, Ankara, Turkey; 121 Bogazici University, Istanbul, Turkey; 122 Istanbul Technical University, Istanbul, Turkey; 123 Institute for Scintillation Materials of National Academy of Science of Ukraine, Kharkov, Ukraine; 124 National Scientific Center, Kharkov Institute of Physics and Technology, Kharkov, Ukraine; 125 University of Bristol, Bristol, UK; 126 Rutherford Appleton Laboratory, Didcot, UK; 127 Imperial College, London, UK; 128 Brunel University, Uxbridge, UK; 129 Baylor University, Waco, USA; 130 The University of Alabama, Tuscaloosa, USA; 131 Boston University, Boston, USA; 132 Brown University, Providence, USA; 133 University of California, Davis, Davis, USA; 134 University of California, Los Angeles, USA; 135 University of California, Riverside, Riverside, USA; 136 University of California, San Diego, La Jolla, USA; 137 Department of Physics, University of California, Santa Barbara, Santa Barbara, USA; 138 California Institute of Technology, Pasadena, USA; 139 Carnegie Mellon University, Pittsburgh, USA; 140 University of Colorado Boulder, Boulder, USA; 141 Cornell University, Ithaca, USA; 142 Fairfield University, Fairfield, USA; 143 Fermi National Accelerator Laboratory, Batavia, USA; 144 University of Florida, Gainesville, USA; 145 Florida International University, Miami, USA; 146 Florida State University, Tallahassee, USA; 147 Florida Institute of Technology, Melbourne, USA; 148 University of Illinois at Chicago (UIC), Chicago, USA; 149 The University of Iowa, Iowa City, USA; 150 Johns Hopkins University, Baltimore, USA; 151 The University of Kansas, Lawrence, USA; 152 Kansas State University, Manhattan, USA; 153 Lawrence Livermore National Laboratory, Livermore, USA; 154 University of Maryland, College Park, USA; 155 Massachusetts Institute of Technology, Cambridge, USA; 156 University of Minnesota, Minneapolis, USA; 157 University of Mississippi, Oxford, USA; 158 University of Nebraska-Lincoln, Lincoln, USA; 159 State University of New York at Buffalo, Buffalo, USA; 160 Northeastern University, Boston, USA; 161 Northwestern University, Evanston, USA; 162 University of Notre Dame, Notre Dame, USA; 163 The Ohio State University, Columbus, USA; 164 Princeton University, Princeton, USA; 165 University of Puerto Rico, Mayaguez, USA; 166 Purdue University, West Lafayette, USA; 167 Purdue University Calumet, Hammond, USA; 168 Rice University, Houston, USA; 169 University of Rochester, Rochester, USA; 170 Rutgers, The State University of New Jersey, Piscataway, USA; 171 University of Tennessee, Knoxville, USA; 172 Texas A&M University, College Station, USA; 173 Texas Tech University, Lubbock, USA; 174 Vanderbilt University, Nashville, USA; 175 University of Virginia, Charlottesville, USA; 176 Wayne State University, Detroit, USA; 177 University of Wisconsin-Madison, Madison, WI USA; 178 CERN, 1211 Geneva 23, Switzerland

## Abstract

A search for new phenomena is performed in final states containing one or more jets and an imbalance in transverse momentum in pp collisions at a centre-of-mass energy of 13$\,\text {TeV}$. The analysed data sample, recorded with the CMS detector at the CERN LHC, corresponds to an integrated luminosity of 2.3$\,\text {fb}^{-1}$. Several kinematic variables are employed to suppress the dominant background, multijet production, as well as to discriminate between other standard model and new physics processes. The search provides sensitivity to a broad range of new-physics models that yield a stable weakly interacting massive particle. The number of observed candidate events is found to agree with the expected contributions from standard model processes, and the result is interpreted in the mass parameter space of fourteen simplified supersymmetric models that assume the pair production of gluinos or squarks and a range of decay modes. For models that assume gluino pair production, masses up to 1575 and 975$\,\text {GeV}$ are excluded for gluinos and neutralinos, respectively. For models involving the pair production of top squarks and compressed mass spectra, top squark masses up to 400$\,\text {GeV}$ are excluded.

## Introduction

The standard model (SM) of particle physics is successful in describing a wide range of phenomena, although it is widely believed to be only an effective approximation of a more complete theory that supersedes it at a higher energy scale. Supersymmetry (SUSY) [1–4] is a modification to the SM that extends its underlying space-time symmetry group. For each boson (fermion) in the SM, a fermionic (bosonic) superpartner, which differs in spin by one-half unit, is introduced.

Experimentally, SUSY is testable through the prediction of an extensive array of new observable states (of unknown masses) [5, 6]. In the minimal supersymmetric extension to the SM [6], the gluinos $\widetilde{\mathrm{g}}$, light- and heavy-flavour squarks $\widetilde{\mathrm{q}}, \widetilde{\mathrm{b}}, \widetilde{\mathrm{t}}$, and sleptons $\widetilde{\ell }$ are, respectively, the superpartners to gluons, quarks, and leptons. An extended Higgs sector is also predicted, as well as four neutralino $\widetilde{\chi }^0 _{1,2,3,4}$ and two chargino $\widetilde{\chi }^\pm _{1,2}$ states that arise from mixing between the higgsino and gaugino states, which are the superpartners of the Higgs and electroweak gauge bosons. The assumption of *R*-parity conservation [7] has important consequences for cosmology and collider phenomenology. Supersymmetric particles are expected to be produced in pairs at the LHC, with heavy coloured states decaying, potentially via intermediate SUSY states, to the stable lightest SUSY particle (LSP). The LSP is generally assumed to be the $\widetilde{\chi }^{0}_{1}$, which is weakly interacting and massive. This SUSY particle is considered to be a candidate for dark matter (DM) [8], the existence of which is supported by astrophysical data [9]. Hence, a characteristic signature of R-parity-conserving coloured SUSY production at the LHC is a final state containing an abundance of jets, possibly originating from top or bottom quarks, accompanied by a significant transverse momentum imbalance, ${\vec {p}}_{\mathrm {T}}^{\text {miss}}$.

The proposed supersymmetric extension of the SM is also compelling from a theoretical perspective, as the addition of superpartners to SM particles can modify the running of the gauge coupling constants such that their unification can be achieved at a high energy scale [10–12]. A more topical perspective, given the recently discovered Higgs boson [13–15], is the possibility that scale-dependent radiative corrections to the Higgs boson mass from loop processes can be largely cancelled through the introduction of superpartners, thus alleviating the gauge hierarchy problem [16, 17]. Alternatively, these radiative corrections can be accommodated through an extreme level of fine tuning of the bare Higgs boson mass. A “natural” solution from SUSY, with minimal fine-tuning, implies that the masses of the $\widetilde{\chi }^{0}_{1}$, third-generation squarks, and the gluino are at or near the electroweak scale [18].

The lack of evidence to date for SUSY has also focused attention on regions of the natural parameter space with sparse experimental coverage, such as phenomenologically well motivated models for which both the $\widetilde{\mathrm{t}}$ and the $\widetilde{\chi }^{0}_{1}$ are light and nearly degenerate in mass [19–27]. This class of models, with “compressed” mass spectra, typically yield SM particles with low transverse momenta ($p_{\mathrm{T}}$) from the decays of SUSY particles. Hence, searches rely on the associated production of jets, often resulting from initial-state radiation (ISR), to achieve experimental acceptance.

This paper presents an inclusive search for new-physics phenomena in hadronic final states with one or more energetic jets and an imbalance in ${\vec {p}}_{\mathrm {T}}^{\text {miss}}$, performed in proton–proton (pp) collisions at a centre-of-mass energy $\sqrt{s} = 13\,\text {TeV}$. The analysed data sample corresponds to an integrated luminosity of $2.3 \pm 0.1{\,\text {fb}^{-1}}$ collected by the CMS experiment. Earlier searches using the same technique have been performed in pp collisions at both $\sqrt{s} = 7$ [28–30] and $8\,\text {TeV}$ [31, 32] by the CMS Collaboration. The increase in the centre-of-mass energy of the LHC, from $\sqrt{s} = 8$ to 13$\,\text {TeV}$, provides a unique opportunity to search for the characteristic signatures of new physics at the $\,\text {TeV}$ scale. For example, the increase in $\sqrt{s}$ leads to a factor $\gtrsim$35 increase in the parton luminosity [33] for the pair production of coloured SUSY particles, each of mass 1.5$\,\text {TeV}$, which were beyond the reach of searches performed at $\sqrt{s} = 8\,\text {TeV}$ by the ATLAS [34, 35] (and references therein) and CMS [36–43] Collaborations. Several searches in this final state, interpreted within the context of SUSY, have already provided results with the first data at this new energy frontier [44–50].

Two important features of this search are the application of selection criteria with low thresholds, in order to maximise signal acceptance, and the categorisation of candidate signal events according to multiple discriminating variables for optimal signal extraction over a broad range of models. The search is based on an examination of the number of reconstructed jets per event, the number of these jets identified as originating from bottom quarks, and the scalar and vector $p_{\mathrm{T}}$ sums of these jets. These variables provide sensitivity to the different production mechanisms (squark–squark, squark–gluino, and gluino–gluino) of massive coloured SUSY particles at hadron colliders, third-generation squark signatures, and both large and small mass splittings between the parent SUSY particle and the LSP. However, the search is sufficiently generic and inclusive to provide sensitivity to a wide range of SUSY and non-SUSY models that postulate the existence of a stable, only weakly interacting, massive particle. In addition to the jets+${\vec {p}}_{\mathrm {T}}^{\text {miss}}$ topology, the search considers final states containing a “monojet” topology, which is expected to improve the sensitivity to DM particle production in pp collisions [51, 52].

The dominant background process for a search in all-jet final states is multijet production, a manifestation of quantum chromodynamics (QCD). An accurate estimate of this background is difficult to achieve, given the lack of precise theoretical predictions for the multijet production cross section and kinematic properties. Hence, this search adopts a strategy that employs several variables to reduce the multijet contribution to a low level with respect to other SM backgrounds, rather than estimate a significant contribution with high precision.

The search is built around two variables that are designed to provide robust discrimination against multijet events. A dimensionless kinematic variable $\alpha _{\mathrm {T}}$  [28, 53] is constructed from jet-based quantities and provides discrimination between genuine sources of ${\vec {p}}_{\mathrm {T}}^{\text {miss}}$ from stable, weakly interacting particles such as neutrinos or neutralinos that escape the detector, and instrumental sources such as the mismeasurements of jet energies. The $\Delta \phi ^{*}_\text {min}$  [28] variable exploits azimuthal angular information and also provides strong rejection power against multijet events, including rare energetic events in which neutrinos carry a significant fraction of the energy of a jet due to semileptonic decays of heavy-flavour mesons. Very restrictive requirements on the $\alpha _{\mathrm {T}}$ and $\varDelta \phi ^{*}_\mathrm{{min}}$ variables are employed in this search to ensure a low level of contamination from the multijet background.

The organisation of this paper is as follows. Sections 2 and 3 describe, respectively, the CMS apparatus and the simulated event samples. Sections 4 and 5 describe the event reconstruction and selection criteria used to identify candidate signal events and control region samples. Section 6 provides details on the estimation of the multijet and all other SM backgrounds. Finally, the search results and interpretations, in terms of simplified SUSY models, are described in Sects. 7 and 8, and summarised in Sect. 9.

## The CMS detector

The central feature of the CMS apparatus is a superconducting solenoid of 6 $\text {m}$ internal diameter, providing an axial magnetic field of 3.8 $\text {T}$. The bore of the solenoid is instrumented with several particle detection systems. A silicon pixel and strip tracker measures charged particles within the pseudorapidity range $|\eta | < 2.5$. A lead tungstate crystal electromagnetic calorimeter (ECAL), and a brass and scintillator hadron calorimeter (HCAL), each composed of a barrel and two endcap sections, extend over a range $|\eta | < 3.0$. Outside the bore of the solenoid, forward calorimeters extend the coverage to $|\eta | < 5.0$, and muons are measured within $|\eta | < 2.4$ by gas-ionisation detectors embedded in the steel flux-return yoke outside the solenoid. A two-tier trigger system selects pp collision events of interest. The first level of the trigger system, composed of custom hardware processors, uses information from the calorimeters and muon detectors to select the most interesting events in a fixed time interval of less than 4 ${\upmu }$s. The high-level trigger processor farm further decreases the event rate from around 100 $\text {kHz}$ to less than 1 $\text {kHz}$, before data storage. The CMS detector is nearly hermetic, which allows for momentum balance measurements in the plane transverse to the beam axis. A more detailed description of the CMS detector, together with a definition of the coordinate system used and the relevant kinematic variables, can be found in Ref. [54].

## Simulated event samples

The search relies on multiple event samples, in data or generated from Monte Carlo (MC) simulations, to estimate the contributions from SM backgrounds, as described in Sect. 5. The SM backgrounds for the search are QCD multijet, top quark-antiquark ($\mathrm{t}\overline{\mathrm{t}}$), and single top production, and the associated production of jets and a vector boson (W, $\mathrm{Z}\rightarrow \nu \overline{\nu }$). Residual contributions from other processes, such as WW, WZ, ZZ (diboson) production and the associated production of $\mathrm{t}\overline{\mathrm{t}}$ and a W or Z boson, are also considered. Other processes, such as Drell–Yan ($\mathrm{q}\bar{\mathrm{q}} \rightarrow \mathrm{Z}/\gamma ^* \rightarrow \ell ^+\ell ^-$) and $\gamma + \text {jets}$ production, are also relevant for some control regions, defined in Sect. 5.5.

The MadGraph 5_amc@nlo 2.2.2 [55] event generator code is used at leading-order (LO) accuracy to produce samples of $\text {W} + \text {jets}$, $\mathrm{Z}+ \text {jets}$, $\gamma + \text {jets}$, $\mathrm{t}\overline{\mathrm{t}}$, and multijet events. The same code is used at next-to-leading-order (NLO) accuracy to generate samples of single top quarks (both *s*- and *t*-channel production), WZ, ZZ, $\mathrm{t}\overline{\mathrm{t}} \text {W}$, and $\mathrm{t}\overline{\mathrm{t}} \mathrm{Z}$ events. The NLO powheg v2 [56, 57] generator is used to describe WW events and the tW-channel production of single top quark events. The simulated samples are normalised according to production cross sections that are calculated with NLO and next-to-NLO precision [55, 57–62], or with LO precision in the case of multijet and $\gamma + \text {jets}$ production. The Geant4  [63] package is used to simulate the detector response.

Event samples for signal models involving gluino or squark pair production in association with up to two additional partons are generated at LO with MadGraph 5_amc@nlo, and the decay of the SUSY particles is performed with pythia 8.205 [64]. Inclusive, process-dependent, signal production cross sections are calculated with NLO plus next-to-leading-logarithmic (NLL) accuracy [33, 65–69]. The theoretical systematic uncertainties are typically dominated by the parton density function (PDF) uncertainties, evaluated using the CTEQ6.6 [70] and MSTW2008 [71] PDFs. The detector response for signal models is provided by the CMS fast simulation package [72].

The NNPDF3.0 LO and NLO [73] parton distribution functions (PDFs) are used, respectively, with the LO and NLO generators described above. The pythia program with the CUETP8M1 underlying event tune [74] is used to describe parton showering and hadronisation for all simulated samples. To model the effects of multiple pp collisions within the same or neighbouring bunch crossings (pileup), all simulated events are generated with a nominal distribution of pp interactions per bunch crossing and then reweighted to match the pileup distribution as measured in data. On average, approximately fifteen different pp collisions, identifiable via their primary interaction vertex, are reconstructed per event. Finally, (near-unity) corrections to the normalisation of the simulated samples for the $\gamma + \text {jets}$, $\text {W} (\rightarrow \mu \nu ) + \text {jets}$, $\mathrm{t}\overline{\mathrm{t}}$, $\mathrm{Z}(\rightarrow \mu \mu ) + \text {jets}$, and $\mathrm{Z}(\rightarrow \nu \overline{\nu }) + \text {jets}$ processes are derived using data sidebands to the control regions.

## Event reconstruction

Global event reconstruction is provided by the particle flow (PF) algorithm [75, 76], designed to identify each particle using an optimised combination of information from all detector systems. In this process, the identification of the particle type (photon, electron, muon, charged hadron, neutral hadron) plays an important role in the determination of the particle direction and energy.

Among the vertices reconstructed within 24 (2) $\text {cm}$ of the detector centre parallel (perpendicular) to the beam axis, the primary vertex (PV) is assigned to be the one with the largest sum of charged particle (track) $p_{\mathrm{T}} ^2$ values. Charged-particle tracks associated with reconstructed vertices from pileup events are not considered by the PF algorithm as part of the global event reconstruction.

Photon candidates [77] are identified as ECAL energy clusters not linked to the extrapolation of any track to the ECAL. The energy of photons is directly obtained from the ECAL measurement, corrected for contributions from pileup events. Various quality-related criteria must be satisfied in order to identify photons with high efficiency while minimising the misidentification of electrons and associated bremsstrahlung, jets, or ECAL noise as photons. The criteria include the following: the shower shape of the energy deposition in the ECAL must be consistent with that expected from a photon, the energy detected in the HCAL behind the photon shower must not exceed 5% of the photon energy, and no matched hits in the pixel tracker must be found.

Electron candidates [78] are identified as a track associated with an ECAL cluster compatible with the track trajectory, as well as additional ECAL energy clusters from potential bremsstrahlung photons emitted as the electron traverses material of the silicon tracker. The energy of electrons is determined from a combination of the track momentum at the main interaction vertex, the corresponding ECAL cluster energy, and the energy sum of all bremsstrahlung photons associated with the track. The quality criteria required for electrons are similar to those for photons, with regards to the ECAL shower shape and the relative contributions to the total energy deposited in the ECAL and HCAL. Additional requirements are also made on the associated track, which consider the track quality, energy-momentum matching, and compatibility with the PV in terms of the transverse and longitudinal impact parameters.

Muon candidates [79] are identified as a track in the silicon tracker consistent with either a track or several hits in the muon system. The track and hit parameters must satisfy various quality-related criteria, described in Ref. [79]. The energy of muons is obtained from the corresponding track momentum.

Charged hadrons are identified as tracks not classified as either electrons or muons. The energy of charged hadrons is determined from a combination of the track momentum and the corresponding ECAL and HCAL energies, corrected for contributions from pileup events and the response function of the calorimeters to hadronic showers. Neutral hadrons are identified as HCAL energy clusters not linked to any charged-hadron trajectory, or as ECAL and HCAL energy excesses with respect to the expected charged-hadron energy deposit. The energy of neutral hadrons is obtained from the corresponding corrected ECAL and HCAL energy.

Photons are required to be isolated from other activity in the event, such as charged and neutral hadrons, within a cone $\varDelta R = \sqrt{\smash [b]{(\varDelta \phi )^2 + (\varDelta \eta )^2}} = 0.3$ around the photon trajectory, corrected for contributions from pileup events and the photon itself. Electrons and muons are also required to be isolated from other reconstructed particles in the event, primarily to suppress background contributions from semileptonic heavy-flavour decays in multijet events. The isolation $I^\text {mini}_\text {rel}$ is defined as the scalar $p_{\mathrm{T}}$ sum of all charged and neutral hadrons, and photons, within a cone around the lepton direction, divided by the lepton $p_{\mathrm{T}}$. The “mini” cone radius is dependent on the lepton $p_{\mathrm{T}}$, primarily to identify with high efficiency the collimated daughter particles of semileptonically decaying Lorentz-boosted top quarks, according to the following: $R = 0.2$ and 0.05 for, respectively, $p_{\mathrm{T}} < 50\,\text {GeV}$ and $p_{\mathrm{T}} > 200\,\text {GeV}$, and $R = 10\,\text {GeV}/ p_{\mathrm{T}}$ for $50< p_{\mathrm{T}} < 200\,\text {GeV}$. The variable $I^\text {mini}_\text {rel}$ excludes contributions from the lepton itself and pileup events. The isolation for electrons and muons is required to satisfy, respectively, $I^\text {mini}_\text {rel} < 0.1$ and 0.2 for the signal region and nonleptonic control sample selection criteria. A tighter definition of muon isolation $I^{\mu }_\text {rel}$ is used for the definition of control regions that are required to contain at least one muon. The variable $I^{\mu }_\text {rel}$ is determined identically to $I^\text {mini}_\text {rel}$ except that a cone of fixed radius $R = 0.4$ is assumed.

Electron and muon candidates identified by the PF algorithm that do not satisfy the quality criteria or the $I^\text {mini}_\text {rel}$ isolation requirements described above, as well as charged hadrons, are collectively labelled as “single isolated tracks” if they are isolated from neighbouring tracks associated to the PV. The isolation $I^\text {track}_\text {rel}$ is defined as the scalar $p_{\mathrm{T}}$ sum of tracks (excluding the track under consideration) within a cone $\varDelta R < 0.3$ around the track direction, divided by the track $p_{\mathrm{T}}$. The requirement $I^\text {track}_\text {rel} < 0.1$ is imposed.

Jets are clustered from the PF candidate particles with the infrared- and collinear-safe anti-$k_t$ algorithm [80], operated with a distance parameter of 0.4. The jet momentum is determined as the vectorial sum of all particle momenta in the jet, and is found in the simulation to be within 5–10% of its true momentum over the whole $p_{\mathrm{T}}$ spectrum and detector acceptance. Jet energy corrections, to account for pileup [81] and to establish a uniform relative response in $\eta$ and a calibrated absolute response in $p_{\mathrm{T}}$, are derived from the simulation, and are confirmed with in situ measurements using the energy balance in dijet and photon$+$jet events [82]. The jet energy resolution is typically 15% at 10$\,\text {GeV}$, 8% at 100$\,\text {GeV}$, and 4% at 1$\,\text {TeV}$, compared to about 40, 12, and 5% obtained when the calorimeters alone are used for jet clustering. All jets are required to satisfy loose requirements on the relative composition of their particle constituents to reject noise in the calorimeter systems or failures in event reconstruction.

Jets are identified as originating from b quarks using the combined secondary vertex algorithm [83]. Control regions in data [84] are used to measure the probability of correctly identifying jets as originating from b quarks (b tagging efficiency), and the probability of misidentifying jets originating from light-flavour partons (u, d, s quarks or gluons) or a charm quark as a b-tagged jet (the light-flavour and charm mistag probabilities). A working point is employed that yields a b tagging efficiency of 65%, and charm and light-flavour mistag probabilities of approximately 12 and 1%, respectively, for jets with $p_{\mathrm{T}}$ that is typical of $\mathrm{t}\overline{\mathrm{t}}$ events.

An estimator of ${\vec {p}}_{\mathrm {T}}^{\text {miss}}$ is given by the projection on the plane perpendicular to the beams of the negative vector sum of the momenta of all candidate particles in an event [85], as determined by the PF algorithm. Its magnitude is referred to as $E_{\mathrm {T}}^{\text {miss}}$.

## Event selection

The kinematic selection criteria used to define the signal region, containing a sample of candidate signal events, as well as a number of control regions in data, are described below. The criteria are based on the particle candidates defined by the event reconstruction algorithms described in Sect. 4.

### Common preselection criteria

A number of beam- and detector-related effects, such as beam halo, reconstruction failures, spurious detector noise, or event misreconstruction due to detector inefficiencies, can lead to events with anomalous levels of activity. These rare events, which can exhibit large values of $E_{\mathrm {T}}^{\text {miss}}$, are rejected with high efficiency by applying a range of dedicated vetoes [85, 86].

In order to suppress SM processes with genuine ${\vec {p}}_{\mathrm {T}}^{\text {miss}}$ from neutrinos, events containing an isolated electron or muon that satisfies $p_{\mathrm{T}} > 10\,\text {GeV}$ and $|\eta | < 2.5$ are vetoed. Events containing an isolated photon with $p_{\mathrm{T}} > 25\,\text {GeV}$ and $|\eta | < 2.5$ are also vetoed, in order to select only multijet final states. Furthermore, events containing a single isolated track satisfying $p_{\mathrm{T}} > 10\,\text {GeV}$ and $|\eta | < 2.5$ are vetoed in order to reduce the background contribution from final states containing hadronically decaying tau leptons.

Each jet $\mathrm{{j}}_i$ considered by this search is required to satisfy $p_{\mathrm{T}} ^{\mathrm{{j}}_i} > 40\,\text {GeV}$ and $|\eta ^{\mathrm{{j}}_i}| < 3$. The number of jets within this experimental acceptance is labelled henceforth as $n_{\text {jet}}$. The highest $p_{\mathrm{T}}$ jet in the event is required to have $p_{\mathrm{T}} ^{\mathrm{{j}_1}} > 100\,\text {GeV}$ and $|\eta ^{\mathrm{{j}_1}} | < 2.5$. The second-highest $p_{\mathrm{T}}$ jet in the event is used to categorise events, as described in Sect. 5.2. If the jet satisfies $p_{\mathrm{T}} ^{\mathrm{{j}_2}} > 100\,\text {GeV}$, then this category of events is labelled “symmetric” and targets primarily topologies resulting from pair-produced SUSY particles. If the jet satisfies $40< p_{\mathrm{T}} ^{\mathrm{{j}_2}} < 100\,\text {GeV}$ then the event is labelled as “asymmetric,” and if there exists no second jet with $p_{\mathrm{T}} ^{\mathrm{{j}_2}} > 40\,\text {GeV}$, the event is labelled monojet. The asymmetric and monojet topologies target models involving the direct production of stable, weakly interacting, massive particles. The mass scale of the physics processes being probed is characterised by the scalar $p_{\mathrm{T}}$ sum of the jets, defined as $H_{\mathrm {T}} = \sum _{i=1}^{n_{\text {jet}}} p_{\mathrm{T}} ^{\,\mathrm{{j}}_i}$. The magnitude of the vector ${\vec {p}}_{\mathrm {T}}$ sum of these jets, defined by $H_{\mathrm {T}}^{\text {miss}} = |\sum _{{i}=1}^{n_{\text {jet}}} {\vec {p}}_{\mathrm {T}} ^{\,\mathrm{{j}}_i} |$, is used to identify events with significant imbalance in ${\vec {p}}_{\mathrm {T}}^{\text {miss}}$. Events are vetoed if any jet satisfies $p_{\mathrm{T}} > 40\,\text {GeV}$ and $|\eta | > 3$ to ensure that jets reconstructed in the forward regions of the detector do not contribute significantly to $H_{\mathrm {T}}^{\text {miss}}$.

The dimensionless variable $H_{\mathrm {T}}^{\text {miss}}/ E_{\mathrm {T}}^{\text {miss}}$ is used to remove events that contain several jets with transverse momenta below the jet $p_{\mathrm{T}}$ thresholds but an appreciable vector $p_{\mathrm{T}}$ sum so as to contribute significantly to $H_{\mathrm {T}}^{\text {miss}}$ relative to $E_{\mathrm {T}}^{\text {miss}}$. This background is typical of multijet events, which is suppressed by requiring $H_{\mathrm {T}}^{\text {miss}}/ E_{\mathrm {T}}^{\text {miss}} < 1.25$. The requirement is imposed as part of the common preselection criteria used to define all control samples to minimise potential systematic biases associated with the simulation modelling for this variable. A high efficiency is maintained for SM or new-physics processes that produce unobserved particles, which are characterised by large values of ${\vec {p}}_{\mathrm {T}}^{\text {miss}}$ and values of $H_{\mathrm {T}}^{\text {miss}}/ E_{\mathrm {T}}^{\text {miss}}$ close to unity.

Significant jet activity and ${\vec {p}}_{\mathrm {T}}^{\text {miss}}$ in the event is ensured by requiring $H_{\mathrm {T}} > 200\,\text {GeV}$ and $H_{\mathrm {T}}^{\text {miss}} > 130\,\text {GeV}$, respectively. These requirements complete the common preselection criteria, summarised in Table 1, used to define a sample of all-jet events characterised by high jet activity and appreciable ${\vec {p}}_{\mathrm {T}}^{\text {miss}}$.

**Table 1:** Summary of the event selection criteria and categorisation used to define the signal and control regions

Common preselection	
$E_{\mathrm {T}}^{\text {miss}}$ quality	Filters related to beam and instrumental effects, and reconstruction failures
Lepton/photon vetoes	$p_{\mathrm{T}} > 10,\, 10,\, 25\,\text {GeV}$ for isolated tracks, leptons, photons (respectively) and $|\eta | < 2.5$
Jet $\mathrm{{j}}_i$ acceptance	Consider each jet $\mathrm{{j}}_i$ that satisfies $p_{\mathrm{T}} ^{\mathrm{{j}}_i} > 40\,\text {GeV}$ and $|\eta ^{\mathrm{{j}_1}} | < 3$
Jet $\mathrm{{j}_1}$ acceptance	$p_{\mathrm{T}} ^{\mathrm{{j}_1}} > 100\,\text {GeV}$ and $|\eta ^{\mathrm{{j}_1}} | < 2.5$
Jet $\mathrm{{j}_2}$ acceptance	$p_{\mathrm{T}} ^{\mathrm{{j}_2}} < 40\,\text {GeV}$ (monojet), $40< p_{\mathrm{T}} ^{\mathrm{{j}_2}} < 100\,\text {GeV}$ (asymmetric), $p_{\mathrm{T}} ^{\mathrm{{j}_2}} > 100\,\text {GeV}$ (symmetric)
Forward jet veto	Veto events containing a jet satisfying $p_{\mathrm{T}} > 40\,\text {GeV}$ and $|\eta | > 3$
Jets below threshold	$H_{\mathrm {T}}^{\text {miss}}/ E_{\mathrm {T}}^{\text {miss}} < 1.25$
Energy sums	$H_{\mathrm {T}} > 200\,\text {GeV}$ and $H_{\mathrm {T}}^{\text {miss}} > 130\,\text {GeV}$
Event categorisation	
$n_{\text {jet}}$	1 (monojet), 2, 3, 4, $\ge$5 (asymmetric), 2, 3, 4, $\ge$5 (symmetric)
$n_{\text {b}}$	0, 1, 2, $\ge$3 ($n_{\text {b}} \le n_{\text {jet}}$)
$H_{\mathrm {T}}$ ($\text {GeV}$ )	200, 250, 300, 350, 400, 500, 600, ${>}800\,\text {GeV}$ (bins can be dropped/merged *vs.* $n_{\text {jet}}$, Table 2)
Signal region (SR)	Preselection +
QCD multijet rejection	$\alpha _{\mathrm {T}} > 0.65$, 0.60, 0.55, 0.53, 0.52, 0.52, 0.52 (mapped to $H_{\mathrm {T}}$ bins in range $200< H_{\mathrm {T}} < 800\,\text {GeV}$)
QCD multijet rejection	$\Delta \phi ^{*}_\text {min} > 0.5$ ($n_{\text {jet}} \ge 2$) or $\varDelta \phi ^{*_{\, 25}}_\mathrm{{min}} > 0.5$ ($n_{\text {jet}} = 1$)
Control regions (CR)	Preselection +
Multijet-enriched	SR + $H_{\mathrm {T}}^{\text {miss}}/E_{\mathrm {T}}^{\text {miss}} > 1.25$ (inverted)
$\gamma + \text {jets}$	1$\gamma$ with $p_{\mathrm{T}} > 200\,\text {GeV}$, $|\eta | < 1.45$, $\Delta R(\gamma ,\mathrm{{j}}_i) > 1.0$, $H_{\mathrm {T}} > 400\,\text {GeV}$, same $\alpha _{\mathrm {T}}$ req. as SR
$\mu + \text {jets}$	1$\mu$ with $p_{\mathrm{T}} > 30\,\text {GeV}$, $|\eta | < 2.1$, $I^{\mu }_\text {rel} < 0.1$, $\Delta R(\mu ,\mathrm{{j}}_i) > 0.5$, $30< m_\mathrm{{T}}({\vec {p}}_{\mathrm {T}} ^\mu ,{\vec {p}}_{\mathrm {T}}^{\text {miss}}) < 125\,\text {GeV}$
$\mu ^{\pm }\mu ^{\mp } + \text {jets}$	2$\mu$ with $p_{\mathrm{T}} > 30\,\text {GeV}$, $|\eta | < 2.1$, $I^{\mu }_\text {rel} < 0.1$, $\Delta R(\mu _{1,2},\mathrm{{j}}_i) > 0.5$, $|m_{\mu \mu } - m_\text {Z} | < 25\,\text {GeV}$

### Event categorisation

Events selected by the common preselection criteria are categorised according to $n_{\text {jet}}$, the number of b-tagged jets $n_{\text {b}}$, and $H_{\mathrm {T}}$. Nine categories in $n_{\text {jet}}$ are employed: the monojet topology ($n_{\text {jet}} = 1$) and four $n_{\text {jet}}$ bins (2, 3, 4, $\ge$5) for each of the asymmetric and symmetric topologies. Events are also categorised by $n_{\text {b}}$ (0, 1, 2, $\ge$3), where $n_{\text {b}}$ is bounded from above by $n_{\text {jet}}$, resulting in 32 categories in terms of both $n_{\text {jet}}$ and $n_{\text {b}}$. For each ($n_{\text {jet}}$, $n_{\text {b}}$) category, events are binned according to $H_{\mathrm {T}}$: four 50$\,\text {GeV}$ bins at low jet activity in the range $200< H_{\mathrm {T}} < 400\,\text {GeV}$, two 100$\,\text {GeV}$ bins in the range $400< H_{\mathrm {T}} < 600\,\text {GeV}$, one bin covering the region $600< H_{\mathrm {T}} < 800\,\text {GeV}$, and a final open bin for $H_{\mathrm {T}} > 800\,\text {GeV}$. These categorisations are summarised in Table 1. The $H_{\mathrm {T}}$ binning scheme is adapted independently per ($n_{\text {jet}}$, $n_{\text {b}}$) category by removing or merging bins to satisfy a threshold on the minimum number of data events in the control regions, which are used to estimate SM backgrounds, provide checks, and validate assumptions within the methods. The lower bounds of the first and final (open) bins in $H_{\mathrm {T}}$ are summarised in Table 2. In summary, the search employs a categorisation scheme for events that results in 191 bins, defined in terms of $n_{\text {jet}}$, $n_{\text {b}}$, and $H_{\mathrm {T}}$.

### Signal region

For events satisfying the common preselection criteria described above, the multijet background dominates over all other SM backgrounds. Several variables are employed to reduce the multijet contribution to a low level with respect to other SM backgrounds.

The dimensionless kinematic variable $\alpha _{\mathrm {T}}$  [28, 53], defined in Eq. (2) below, is used to provide discrimination against multijet events that do not contain significant ${\vec {p}}_{\mathrm {T}}^{\text {miss}}$ or that contain large ${\vec {p}}_{\mathrm {T}}^{\text {miss}}$ only because of $p_{\mathrm{T}}$ mismeasurements, while retaining sensitivity to new-physics events with significant ${\vec {p}}_{\mathrm {T}}^{\text {miss}}$. The $\alpha _{\mathrm {T}}$ variable depends solely on the transverse component of jet four-momenta and is intrinsically robust against the presence of jet energy mismeasurements in multijet systems. For events containing only two jets, $\alpha _{\mathrm {T}}$ is defined as $\alpha _{\mathrm {T}} = E_{\mathrm {T}} ^{\mathrm{{j}}_2}/M_\mathrm{{T}}$, where $E_{\mathrm {T}} = E\sin \theta$, where *E* is the energy of the jet and $\theta$ is its polar angle with respect to the beam axis, $E_{\mathrm {T}} ^{\mathrm{{j}}_2}$ is the transverse energy of the jet with smaller $E_{\mathrm {T}}$, and $M_\mathrm{{T}}$ is the transverse mass of the dijet system, defined as:

**Table 2:** Summary of the lower bounds of the first and final bins in $H_{\mathrm {T}}$ [$\text {GeV}$ ] (the latter in parentheses) as a function of $n_{\text {jet}}$ and $n_{\text {b}}$

$n_{\text {jet}} {\backslash } n_{\text {b}}$	0	1	2	$\ge$3
Monojet				
1	200 (600)	200 (500)	–	–
Asymmetric				
2	200 (600)	200 (500)	200 (400)	–
3	200 (600)	200 (600)	200 (500)	200 (300)
4	200 (600)	200 (600)	200 (600)	250 (400)
$\ge$5	250 (600)	250 (600)	250 (600)	300 (500)
Symmetric				
2	200 (800)	200 (800)	200 (600)	–
3	200 (800)	250 (800)	250 (800)	– (250)
4	300 (800)	300 (800)	300 (800)	300 (800)
$\ge$5	350 (800)	350 (800)	350 (800)	350 (800)

1 $$\begin{aligned} M_\mathrm{{T}} = \sqrt{ \left( \sum _{i=1,2} E_{\mathrm {T}} ^{\mathrm{{j}}_i} \right) ^2 - \left( \sum _{i=1,2} p_x^{\mathrm{{j}}_i} \right) ^2 - \left( \sum _{i=1,2} p_y^{\mathrm{{j}}_i} \right) ^2}, \end{aligned}$$

where $E_{\mathrm {T}} ^{\mathrm{{j}}_i}$, $p_x^{\mathrm{{j}}_i}$, and $p_y^{\mathrm{{j}}_i}$ are, respectively, the transverse energy, and the *x* and *y* components of the transverse momentum of jet $\mathrm{{j}}_i$. For a perfectly measured dijet event with $E_{\mathrm {T}} ^{\mathrm{{j}_1}} = E_{\mathrm {T}} ^{\mathrm{{j}_2}}$ and back-to-back jets ($\Delta \phi = \pi$), and in the limit in which the momentum of each jet is large compared with its mass, the value of $\alpha _{\mathrm {T}}$ is 0.5. For an imbalance in the $E_{\mathrm {T}}$ of back-to-back jets, $\alpha _{\mathrm {T}}$ is reduced to a value <0.5, which gives the variable its intrinsic robustness. Values significantly greater than 0.5 are observed when the two jets are not back-to-back and recoil against ${\vec {p}}_{\mathrm {T}}^{\text {miss}}$ from weakly interacting particles that escape the detector.

The definition of the $\alpha _{\mathrm {T}}$ variable can be generalised for events with more than two jets [28]. The mass scale for any process is characterised through the scalar sum of the jet transverse energies, defined as $\mathcal {E}_\mathrm {T} = \sum _{i=1}^{N_\text {jet}} E_{\mathrm {T}} ^{\mathrm{{j}}_i}$, where $N_\text {jet}$ is the number of jets with $E_{\mathrm {T}}$ above a predefined threshold. (The definition of $\mathcal {E}_\mathrm {T}$ should be contrasted with that of $H_{\mathrm {T}}$, the scalar $p_{\mathrm{T}}$ sum of the jets.) For events with three or more jets, a pseudo-dijet system is formed by combining the jets in the event into two pseudo-jets. The $\mathcal {E}_\mathrm {T}$ for each of the two pseudo-jets is given by the scalar $E_{\mathrm {T}}$ sum of its contributing jets. The combination chosen is the one that minimises $\Delta \mathcal {E}_\mathrm {T}$, defined as the difference between these sums for the two pseudo-jets. This clustering criterion assumes a balanced-event hypothesis, which provides strong separation between SM multijet events and events with genuine ${\vec {p}}_{\mathrm {T}}^{\text {miss}}$. The $\alpha _{\mathrm {T}}$ definition can be generalised to:

2 $$\begin{aligned} \alpha _{\mathrm {T}} = \frac{1}{2} \frac{\mathcal {E}_\mathrm {T}- \Delta \mathcal {E}_\mathrm {T} }{\sqrt{(\mathcal {E}_\mathrm {T})^2 - (H_{\mathrm {T}}^{\text {miss}})^2}}. \end{aligned}$$

When jet energies are mismeasured, or there are neutrinos from heavy-flavour quark decays, the magnitudes of $H_{\mathrm {T}}^{\text {miss}}$ and $\Delta \mathcal {E}_\mathrm {T}$ are highly correlated. This correlation is much weaker for *R*-parity-conserving SUSY events, where each of the two decay chains produces an undetected LSP.

Multijet events populate the region $\alpha _{\mathrm {T}} < 0.5$ and the $\alpha _{\mathrm {T}}$ distribution is characterised by a sharp edge at 0.5, beyond which the multijet event yield falls by several orders of magnitude. Multijet events with extremely rare but large stochastic fluctuations in the calorimetric measurements of jet energies can lead to values of $\alpha _{\mathrm {T}}$ slightly above 0.5. The edge at 0.5 sharpens with increasing $H_{\mathrm {T}}$ for events containing at least three jets, primarily due to a corresponding increase in the average jet energy and consequently a (relative) improvement in the jet energy resolution.

For events containing at least two jets, thresholds on the minimum allowed $\alpha _{\mathrm {T}}$ values are applied independent of $n_{\text {jet}}$ and $n_{\text {b}}$ but dependent on $H_{\mathrm {T}}$, for events that satisfy $200< H_{\mathrm {T}} < 800\,\text {GeV}$. The $\alpha _{\mathrm {T}}$ thresholds vary between 0.65 and 0.52 for, respectively, the regions $200< H_{\mathrm {T}} < 250\,\text {GeV}$ and $400< H_{\mathrm {T}} < 800\,\text {GeV}$. No requirement on $\alpha _{\mathrm {T}}$ is made for the region $H_{\mathrm {T}} > 800\,\text {GeV}$. The thresholds employed are summarised in Table 1. The $\alpha _{\mathrm {T}}$ thresholds are motivated both by the trigger conditions used to record the candidate signal events, described below, and by simulation-based studies and estimates of the multijet background derived from data.

An additional variable is based on the minimum azimuthal separation between a jet and the negative vector ${\vec {p}}_{\mathrm {T}}$ sum derived from all other jets in the event [28],

3 $$\begin{aligned} \varDelta \phi ^{*}_{\text {min}} = \min _{\,\forall \, \mathrm{{j}}_k\,\in \, [1,n_{\text {jet}} ]} \varDelta \phi \left( {\vec {p}}_{\mathrm {T}} ^{\,\mathrm{{j}}_k}, \, -\sum _{\begin{array}{c} \mathrm{{j}}_i= 1 \\ \mathrm{{j}}_i \ne \mathrm{{j}}_k \end{array}}^{n_{\text {jet}}} {\vec {p}}_{\mathrm {T}} ^{\,\mathrm{{j}}_i} \right) . \end{aligned}$$

This variable discriminates between final states with genuine ${\vec {p}}_{\mathrm {T}}^{\text {miss}}$, e.g. from the leptonic decay of the W boson, and energetic multijet events that have significant ${\vec {p}}_{\mathrm {T}}^{\text {miss}}$ through jet energy mismeasurements or through the production of neutrinos, collinear with the axis of a jet, from semileptonic heavy-flavour decays. Multijet events populate the region $\varDelta \phi ^{*}_\mathrm{{min}} < 0.5$, with the multijet distribution peaking at a value of zero and falling approximately exponentially over five orders of magnitude to a single-event level at a value of $\varDelta \phi ^{*}_\mathrm{{min}} \approx 0.5$, which is close to the distance parameter value of 0.4 used by the anti-$k_{\mathrm {T}}$ jet clustering algorithm. Events with a genuine source of ${\vec {p}}_{\mathrm {T}}^{\text {miss}}$ exhibit a long tail in $\varDelta \phi ^{*}_\mathrm{{min}}$ with values as large as $\pi$.

**Fig. 1:**
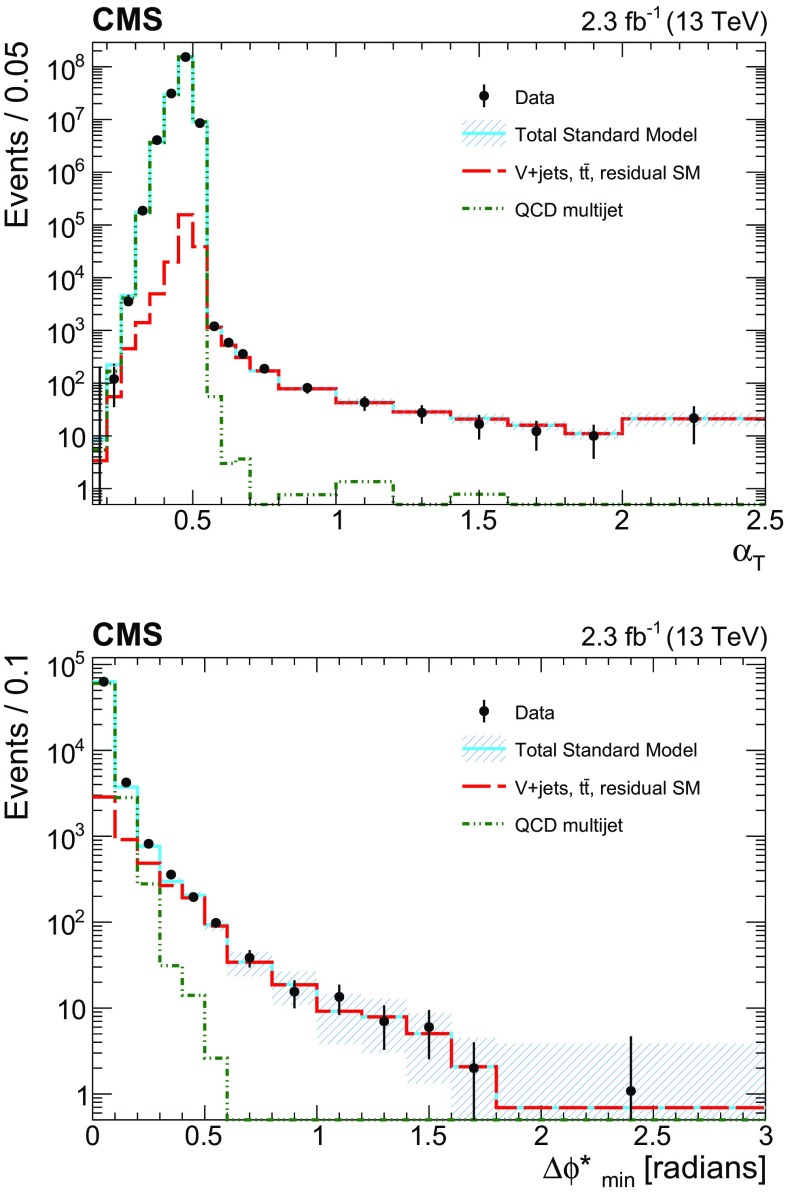
The (*top*) $\alpha _{\mathrm {T}}$ and (*bottom*) $\Delta \phi ^{*}_\text {min}$ distributions observed in data for events that satisfy the selection criteria defined in the text. The statistical uncertainties for the multijet and SM expectations are represented by the hatched areas (visible only for statistically limited bins). The final bin of each distribution contains the overflow events

A requirement of $\varDelta \phi ^{*}_\mathrm{{min}} > 0.5$ is sufficient to effectively suppress the multijet background to a low level while maintaining high efficiency for new-physics signatures. The combined rejection power of the $\alpha _{\mathrm {T}}$ and $\varDelta \phi ^{*}_\mathrm{{min}}$ requirements for the region $200< H_{\mathrm {T}} < 800\,\text {GeV}$ is sufficient to suppress multijet events to the few percent level (and always <10%) with respect to all other SM backgrounds in all $H_{\mathrm {T}}$ bins for all event categories of the signal region. For the region $H_{\mathrm {T}} > 800\,\text {GeV}$, a similar control of the multijet background is achieved solely with the $\varDelta \phi ^{*}_\mathrm{{min}} > 0.5$ requirement.

Figure 1 shows the distributions of $\alpha _{\mathrm {T}}$ and $\varDelta \phi ^{*}_\mathrm{{min}}$ observed in data for events that satisfy the full set of selection criteria used to define the signal region, summarised in Table 1, as well as the following modifications. The $\alpha _{\mathrm {T}}$ and $\varDelta \phi ^{*}_\mathrm{{min}}$ distributions are constructed from events that satisfy $n_{\text {jet}} \ge 2$, $p_{\mathrm{T}} ^{\mathrm{{j}_2}} > 100\,\text {GeV}$, and, respectively, $H_{\mathrm {T}} > 300$ or $800\,\text {GeV}$. In the case of Fig. 1 ($\mathrm{top}$), the events with $\alpha _{\mathrm {T}}$ values greater than 0.55 must fulfill the full set of signal region criteria, including the $\varDelta \phi ^{*}_\mathrm{{min}} > 0.5$ requirement, while the events that satisfy $\alpha _{\mathrm {T}} < 0.55$ are subject to the looser set of common preselection criteria defined in Table 1, excluding the $H_{\mathrm {T}}^{\text {miss}} > 130\,\text {GeV}$ requirement. Hence, Fig. 1 ($\mathrm{top}$) demonstrates the combined performance of several variables that are employed to suppress multijet events. For both distributions, the events are recorded with a set of inclusive trigger conditions that are independent of the $\alpha _{\mathrm {T}}$ and $\varDelta \phi ^{*}_\mathrm{{min}}$ variables. The distributions for the QCD multijet background are determined from simulation while all other SM backgrounds (vector boson production in association with jets, $\mathrm{t}\overline{\mathrm{t}}$, and other residual contributions from rare SM processes) are estimated using a $\mu + \text {jets}$ data control sample, described in Sect. 6.2. The contribution from multijet events is observed to fall by more than five orders of magnitude for both variables.

The $\alpha _{\mathrm {T}}$ and $\varDelta \phi ^{*}_\mathrm{{min}}$ requirements described above, in conjunction with the common preselection requirements $H_{\mathrm {T}}^{\text {miss}} > 130\,\text {GeV}$ and $H_{\mathrm {T}}^{\text {miss}}/ E_{\mathrm {T}}^{\text {miss}} < 1.25$, provide strong rejection power against the multijet background for events that satisfy $n_{\text {jet}} \ge 2$. For monojet events, a modification to the $\varDelta \phi ^{*}_\mathrm{{min}}$ variable, which considers soft jets with $p_{\mathrm{T}} > 25\,\text {GeV}$ ($\varDelta \phi ^{*_{\, 25}}_\mathrm{{min}}$), is utilised. No $\alpha _{\mathrm {T}}$ requirement is imposed, and $\varDelta \phi ^{*_{\, 25}}_\mathrm{{min}} > 0.5$ is sufficient to suppress contributions from multijet events to a negligible level. The aforementioned requirements complete the event selection criteria for the signal region.

Finally, $\varDelta \phi ^{*_{\, 25}}_\mathrm{{min}}$ is also used as a control variable for events that satisfy $n_{\text {jet}} \ge 2$ to identify multijet contributions arising from instrumental effects, such as inefficient detector elements or detector noise. The axis of any jet that satisfies $\varDelta \phi ^{*_{\, 25}}_\mathrm{{min}} < 0.5$ is used to identify localised behaviour in the ($\eta$, $\phi$) plane, which may be indicative of instrumental defects. No significant anomalies are observed in the sample of candidate signal events following the application of the dedicated vetoes described in Sect. 5.1.

Multiple trigger conditions are employed in combination to record candidate signal events. A set of trigger conditions utilise calculations of both $H_{\mathrm {T}}$ and $\alpha _{\mathrm {T}}$ to record events with two or more jets. An event is recorded if it satisfies any of the following pairs of ($H_{\mathrm {T}}$ [$\text {GeV}$ ], $\alpha _{\mathrm {T}}$) thresholds, (200, 0.57), (250, 0.55), (300, 0.53), (350, 0.52), or (400, 0.51), as well as a requirement on the mean value of the two highest $p_{\mathrm{T}}$ jets, $\langle p_{\mathrm{T}} ^{\mathrm{{j}_1}} + p_{\mathrm{T}} ^{\mathrm{{j}_2}}\rangle > 90\,\text {GeV}$. These requirements are collectively labelled as the “$H_{\mathrm {T}}$–$\alpha _{\mathrm {T}}$ ” triggers. In addition, candidate signal events with one or more jets are also recorded if they satisfy the requirements $H_{\mathrm {T}}^{\text {miss}} > 90\,\text {GeV}$ and $E_{\mathrm {T}}^{\text {miss}} > 90\,\text {GeV}$. Finally, for events that satisfy $H_{\mathrm {T}} > 800\,\text {GeV}$, an additional trigger condition, defined by $H_{\mathrm {T}} > 800\,\text {GeV}$, is employed in addition to the $H_{\mathrm {T}}$–$\alpha _{\mathrm {T}}$ trigger requirements to record events characterised by high activity in the calorimeters with high efficiency. The trigger-level jet energies are corrected to account for energy scale and pileup effects. The aforementioned triggers are employed in combination to provide efficiencies at or near 100% for all bins in the signal region.

### Using $H_{\mathrm {T}}^{\text {miss}}$ templates

Following the event selection criteria described above, which provide a sample of candidate signal events with a negligible contribution from multijet events, further discriminating power is required to separate new-physics signatures from the remaining SM backgrounds, which are dominated by the production of $\mathrm{t}\overline{\mathrm{t}}$ or $\text {W} (\rightarrow \ell \nu ) + \text {jets}$ and $\mathrm{Z}(\rightarrow \nu \overline{\nu }) + \text {jets}$ events. As discussed in Sect. 1, the production of coloured SUSY particles with decays to a weakly interacting LSP typically gives rise to a final state with multiple jets and large ${\vec {p}}_{\mathrm {T}}^{\text {miss}}$. The search therefore exploits the $H_{\mathrm {T}}^{\text {miss}}$ variable as an additional discriminant between new-physics and SM processes.

The search relies directly on simulation to determine a template for each ($n_{\text {jet}}$, $n_{\text {b}}$, $H_{\mathrm {T}}$) bin that describes the expected distribution of events as a function of $H_{\mathrm {T}}^{\text {miss}}$. These templates are used by the likelihood function as a model for the data, details of which can be found in Sect. 7. The templates are extensively validated against data in multiple control regions, and these studies are used to establish the uncertainty in the simulation modelling of the $H_{\mathrm {T}}^{\text {miss}}$ variable. The effects of theoretical and experimental uncertainties on the $H_{\mathrm {T}}^{\text {miss}}$ distributions are also studied. Further details can be found in Sect. 6.2.

### Control regions

Four control regions in data are employed to estimate the background contributions from SM processes. The event selection criteria used to define the control regions comprise the common preselection requirements and additional sample-specific requirements, as summarised in Table 1. The first control region comprises a multijet-enriched sample of events, and is defined by the signal region selection criteria and the inverted requirement $H_{\mathrm {T}}^{\text {miss}}/ E_{\mathrm {T}}^{\text {miss}} > 1.25$. The events are recorded with the signal triggers described above, and the sample is used to estimate the multijet background in the signal region. Three additional control regions, defined by inverting one of the photon or lepton vetoes to select samples of $\gamma + \text {jets}$, $\mu + \text {jets}$, or $\mu \mu + \text {jets}$ events, are used to estimate the background contributions from SM processes with final states containing genuine ${\vec {p}}_{\mathrm {T}}^{\text {miss}}$, which are primarily $\mathrm{t}\overline{\mathrm{t}}$, $\text {W} (\rightarrow \ell \nu ) + \text {jets}$, and $\mathrm{Z}(\rightarrow \nu \overline{\nu }) + \text {jets}$.

Additional kinematic requirements are employed to ensure the control samples are enriched in the same SM processes that contribute to background events in the signal region, and are depleted in contributions from multijet production or a wide variety of SUSY models (i.e. so-called signal contamination). The samples are defined, and their events are identically categorised and binned, such that the kinematic properties of events in the control regions and the candidate signal events resemble as closely as possible one another once the photon, muon, or dimuon system is ignored in the calculation of quantities such as $H_{\mathrm {T}}$ and $H_{\mathrm {T}}^{\text {miss}}$. The selections are summarised in Table 1 and described below.

The $\gamma + \text {jets}$ event sample is defined by the common preselection requirements, but the photon veto is inverted and each event is required to contain a single isolated photon, as defined in Sect. 4, that satisfies $p_{\mathrm{T}} > 200\,\text {GeV}$ and $|\eta | < 1.45$ and is well separated from each jet $\mathrm{{j}}_i$ in the event according to $\varDelta R(\gamma ,\mathrm{{j}}_i) > 1.0$. In addition, events must satisfy $H_{\mathrm {T}} > 400\,\text {GeV}$, as well as the same $H_{\mathrm {T}}$-dependent $\alpha _{\mathrm {T}}$ requirements used to define the signal region. The events are recorded using a single-photon trigger condition and the selection criteria result in a trigger efficiency of $\gtrsim$99%.

The $\mu + \text {jets}$ event sample is defined by the common preselection requirements, but the muon veto is inverted and each event is required to contain a single isolated muon, as defined in Sect. 4, that satisfies $p_{\mathrm{T}} > 30\,\text {GeV}$ and $|\eta | < 2.1$ and is well separated from each jet ${\mathrm{{j}}}_i$ in the event according to $\varDelta R(\mu ,{\mathrm{{j}}}_i) > 0.5$. The transverse mass formed by the muon $p_{\mathrm{T}}$ and ${\vec {p}}_{\mathrm {T}}^{\text {miss}}$ system must satisfy $30< m_\mathrm{{T}} < 125\,\text {GeV}$ to select a sample of events rich in W bosons, produced promptly or from the decay of top quarks. The $\mu \mu + \text {jets}$ sample uses a similar set of selection criteria as the $\mu + \text {jets}$ sample, but specifically requires two oppositely charged isolated muons that both satisfy $p_{\mathrm{T}} > 30\,\text {GeV}$ and $|\eta | < 2.1$ and are well separated from the jets in the event ($\varDelta R(\mu _{1,2},\mathrm{{j}}_i) > 0.5$). The muons are also required to have a dilepton invariant mass within a $\pm 25\,\text {GeV}$ window around the nominal mass of the Z boson [9]. For both the muon and dimuon samples, no requirement is made on $\alpha _{\mathrm {T}}$ in order to increase the statistical precision of the predictions from these samples. Both the $\mu + \text {jets}$ and $\mu \mu + \text {jets}$ samples are recorded using a trigger that requires an isolated muon. The selection criteria of the $\mu + \text {jets}$ and $\mu \mu + \text {jets}$ event samples are chosen so that the trigger is maximally efficient, with values of $\sim$90 and $\sim$99%, respectively.

## Estimation of backgrounds

### Multijet background

The signal region is defined in a manner that suppresses the expected contribution from multijet production to a low level with respect to the total expected background from other SM processes for all signal region bins. This is achieved primarily through the application of very tight requirements on the variables $\alpha _{\mathrm {T}}$ and $\varDelta \phi ^{*}_\mathrm{{min}}$, as described in Sect. 5.3, as well as the requirement $H_{\mathrm {T}}^{\text {miss}} / E_{\mathrm {T}}^{\text {miss}} < 1.25$. In this section, we discuss these requirements further, and present the estimate of the multijet background.

The contamination from multijet events in the signal region is estimated using a multijet-enriched data sideband to the signal region, defined by the (inverted) requirement $H_{\mathrm {T}}^{\text {miss}} / E_{\mathrm {T}}^{\text {miss}} > 1.25$. The observed counts in data, categorised according to $n_{\text {jet}}$ and $H_{\mathrm {T}}$, are corrected to account for contamination from nonmultijet SM processes, and the corrected counts $\mathcal {N}^\text {data}(n_{\text {jet}}, H_{\mathrm {T}})$ are assumed to arise solely from QCD multijet production. The nonmultijet processes, which comprise vector boson and $\mathrm{t}\overline{\mathrm{t}}$ production and residual contributions from other SM processes, are estimated using the $\mu + \text {jets}$ control region, as described in Sect. 6.2.

Independent ratios $\mathcal {R}^\mathrm{{QCD}}(n_{\text {jet}}, H_{\mathrm {T}})$ of the number of multijet events that satisfy the requirement $H_{\mathrm {T}}^{\text {miss}} / E_{\mathrm {T}}^{\text {miss}} < 1.25$ to the number that fail this requirement are determined from simulation for events categorised according to $n_{\text {jet}}$ and $H_{\mathrm {T}}$, and inclusively with respect to $n_{\text {b}}$ and $H_{\mathrm {T}}^{\text {miss}}$. The product of each ratio $\mathcal {R}^\mathrm{{QCD}}(n_{\text {jet}}, H_{\mathrm {T}})$ and the corresponding corrected data count $\mathcal {N}^\text {data}(n_{\text {jet}}, H_{\mathrm {T}})$ provides the estimate of the multijet background $\mathcal {P}(n_{\text {jet}}, H_{\mathrm {T}})$. The estimates as a function of $n_{\text {jet}}$, $H_{\mathrm {T}}$, $n_{\text {b}}$, and $H_{\mathrm {T}}^{\text {miss}}$ of the signal region are assumed to factorise as follows:

4 $$\begin{aligned} \mathcal {P}( n_{\text {jet}}, H_{\mathrm {T}}) = \mathcal {N}^\text {data}( n_{\text {jet}}, H_{\mathrm {T}})\; \mathcal {R}^\mathrm{{QCD}}( n_{\text {jet}}, H_{\mathrm {T}}), \end{aligned}$$

5 $$\begin{aligned} \mathcal {P}( n_{\text {jet}}, H_{\mathrm {T}}, n_{\text {b}}, H_{\mathrm {T}}^{\text {miss}}) = \mathcal {P}( n_{\text {jet}}, H_{\mathrm {T}})\; \mathcal {K}_{n_{\text {jet}}, H_{\mathrm {T}}}( n_{\text {b}}, H_{\mathrm {T}}^{\text {miss}}),\nonumber \\ \end{aligned}$$

where $\mathcal {K}_{n_{\text {jet}}, H_{\mathrm {T}}}( n_{\text {b}}, H_{\mathrm {T}}^{\text {miss}})$ are multiplier terms that provide the estimated distribution of events as a function of $n_{\text {b}}$ and $H_{\mathrm {T}}^{\text {miss}}$ while preserving the normalisation $\mathcal {P}( n_{\text {jet}}, H_{\mathrm {T}})$.

**Fig. 2:**
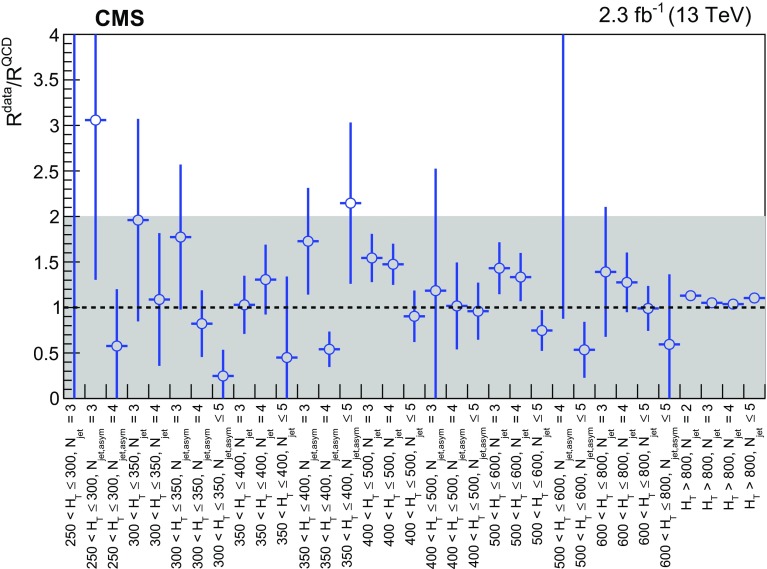
Validation of the ratio $\mathcal {R}^\mathrm{{QCD}}$ determined from simulation in bins of $n_{\text {jet}}$ and $H_{\mathrm {T}}$ [$\text {GeV}$ ] by comparing with an equivalent ratio $\mathcal {R}^\text {data}$ constructed from data in a multijet-enriched sideband to the signal region. A value of unity is expected for the double ratio $\mathcal {R}^\text {data}/\mathcal {R}^\mathrm{{QCD}}$, and the *grey shaded band* represents the assumed systematic uncertainty of 100% in $\mathcal {R}^\mathrm{{QCD}}$

The use of simulation to determine $\mathcal {R}^\mathrm{{QCD}}(n_{\text {jet}}, H_{\mathrm {T}})$ is validated using a multijet-enriched data sideband defined by $\varDelta \phi ^{*}_\mathrm{{min}} < 0.5$. Each ratio $\mathcal {R}^\text {data}(n_{\text {jet}}, H_{\mathrm {T}})$ is constructed from data counts, corrected to account for contributions from nonmultijet processes, and compared with the corresponding ratio $\mathcal {R}^\mathrm{{QCD}}(n_{\text {jet}}, H_{\mathrm {T}})$, determined from simulation, through the double ratio $\mathcal {R}^\text {data}/\mathcal {R}^\mathrm{{QCD}}$, as shown in Fig. 2. The double ratios are statistically compatible with unity across the full phase space of the signal region, including the bins at high $H_{\mathrm {T}}$, which exhibit the highest statistical precision. In addition to statistical uncertainties as large as $\sim$100%, a systematic uncertainty of 100% in $\mathcal {R}^\mathrm{{QCD}}$ is assumed to adequately cover the observed level of agreement for the full signal region phase space.

The distribution of multijet events as a function of $n_{\text {b}}$ and $H_{\mathrm {T}}^{\text {miss}}$, $\mathcal {K}_{n_{\text {jet}}, H_{\mathrm {T}}}( n_{\text {b}}, H_{\mathrm {T}}^{\text {miss}})$, is assumed to be identical to the distribution expected for the nonmultijet backgrounds. This final assumption is based on studies in simulation and is a valid approximation given the magnitude of this background contribution, as well as the magnitude of the statistical and systematic uncertainties in the ratios $\mathcal {R}^\mathrm{{QCD}}(n_{\text {jet}}, H_{\mathrm {T}})$, as described above.

### Backgrounds with genuine $E_{\mathrm {T}}^{\text {miss}}$

Following the suppression of multijet events through the use of the $\alpha _{\mathrm {T}}$ and $\varDelta \phi ^{*}_\mathrm{{min}}$ variables, the dominant nonmultijet backgrounds involve SM processes that produce high-$p_{\mathrm{T}}$ neutrinos in the final state. In events with few jets or few b quark jets, the associated production of W or Z bosons and jets, with the decays $\text {W} ^\pm \rightarrow \ell \nu$ ($\ell =\mathrm {e}$, $\mathrm {\mu }$, $\mathrm {\tau }$) or $\mathrm{Z}\rightarrow \nu \overline{\nu }$, dominate the background counts. For W boson decays that yield an electron or muon (possibly originating from leptonic $\mathrm {\tau }$ decays), the background contributions result from events containing an $\mathrm {e}$ or $\mathrm {\mu }$ that are not rejected by the lepton vetoes. The veto of events containing at least one isolated track further suppresses these backgrounds, including those from single-prong $\tau$-lepton decays. At higher jet or b-quark jet multiplicities, single top quark and $\mathrm{t}\overline{\mathrm{t}}$ production, followed by semileptonic top quark decay, also become an important source of background.

The method to estimate the nonmultijet backgrounds in the signal region relies on the use of a transfer ($\mathcal {T}$) factor determined from simulation that is constructed per bin (in terms of $n_{\text {jet}}$, $n_{\text {b}}$, and $H_{\mathrm {T}}$) per control region. Each $\mathcal {T}$ factor is defined as the ratio of the expected yields in the same ($n_{\text {jet}}$, $n_{\text {b}}$, $H_{\mathrm {T}}$) bins of the signal region $\mathcal {N}^\text {SR}_\text {MC}$ and one of the control regions $\mathcal {N}^\text {CR}_\text {MC}$. The $\mathcal {T}$ factors are used to extrapolate from the event yields observed in each bin of a data control sample $\mathcal {N}^\text {CR}_\text {data}$ to provide an estimate for the background, integrated over $H_{\mathrm {T}}^{\text {miss}}$, from a particular SM process or processes in the corresponding bin of the signal region $\mathcal {N}^\text {SR}_\text {data}$. The superscript SR or CR refers to, respectively, the process or processes being estimated and one of the $\mu + \text {jets}$, $\mu \mu + \text {jets}$, and $\gamma + \text {jets}$ control regions, described in Sect. 5.5. The subscript refers to whether the counts are obtained from data, simulation (“MC”), or an estimate (“pred”).

The method aims to minimise the effects of simulation mismodelling, as many systematic biases in the simulation are expected to largely cancel in the $\mathcal {T}$ factors, given that the events in any given ($n_{\text {jet}}$, $n_{\text {b}}$, $H_{\mathrm {T}}$) bin of the control regions closely mirror those in the corresponding bin in the signal region in terms of the event energy scale, topology, and kinematics. In short, minimal extrapolations are made. Uncertainties in the $\mathcal {T}$ factors are determined from data, as described below.

**Table 3:** Systematic uncertainties (in percent) in the transfer ($\mathcal {T}$) factors used in the method to estimate the SM backgrounds with genuine ${\vec {p}}_{\mathrm {T}}^{\text {miss}}$ in the signal region. The quoted ranges provide representative values of the observed variations as a function of $n_{\text {jet}}$ and $H_{\mathrm {T}}$

Systematic source	Uncertainty in $\mathcal {T}$ factor [%]			
	$\mathcal {T} ^{\text {W}/\mathrm{t}\overline{\mathrm{t}}}_{\mu + \text {jets}}$	$\mathcal {T} ^{\mathrm{Z}\rightarrow \nu \overline{\nu }}_{\mu + \text {jets}}$	$\mathcal {T} ^{\mathrm{Z}\rightarrow \nu \overline{\nu }}_{\mu \mu + \text {jets}}$	$\mathcal {T} ^{\mathrm{Z}\rightarrow \nu \overline{\nu }}_{\gamma + \text {jets}}$
Scale factors (applied to simulation)				
Jet energy scale	<15	<15	<10	<15
b tagging eff and mistag rate	<5	<5	<2	<2
Lepton identification	2–5	2–5	2–5	–
Pileup	<10	<6	<4	<3
Top quark $p_{\mathrm{T}}$	<5	<20	<4	–
Closure tests				
W/Z ratio	–	10–30	–	–
Z/$\gamma$ ratio	–	–	–	10–30
W/$\mathrm{t}\overline{\mathrm{t}}$ composition	10–100	–	–	–
W polarisation	5–50	5–50	–	–
$\alpha _{\mathrm {T}}/\varDelta \phi ^{*}_\mathrm{{min}}$	5–80	5–80	50–80	–

Three independent estimates of the irreducible background of $\mathrm{Z}\rightarrow \nu \overline{\nu }$ + jets events are determined from the $\gamma + \text {jets}$, $\mu \mu + \text {jets}$, and $\mu + \text {jets}$ data control samples. The $\gamma + \text {jets}$ and $\mathrm{Z}\rightarrow \mu \mu$ + jets processes have similar kinematic properties when the photon or muons are ignored [87], albeit different acceptances. In addition, the $\gamma + \text {jets}$ process has a larger production cross section than $\mathrm{Z}\rightarrow \nu \overline{\nu }$ + jets events. The $\mu + \text {jets}$ data sample is used to provide an estimate for both the $\mathrm{Z}\rightarrow \nu \overline{\nu }$ + jets background, as well as the other dominant SM processes, $\mathrm{t}\overline{\mathrm{t}}$ and W boson production (labelled collectively as $\text {W}/\mathrm{t}\overline{\mathrm{t}}$). Residual contributions from all other SM relevant processes, such as single top quark, diboson, and Drell–Yan production, are also included as part of the $\text {W}/\mathrm{t}\overline{\mathrm{t}}$ estimate from the $\mu + \text {jets}$ sample. The definition of the various $\mathcal {T}$ factors used in the search are given below:

6 $$\begin{aligned} \mathcal {N}^{\text {W}/\mathrm{t}\overline{\mathrm{t}}}_\mathrm{{pred}} \,&= \, \mathcal {T} ^{\text {W}/\mathrm{t}\overline{\mathrm{t}}}_{\mu + \text {jets}} \; \mathcal {N}^{\mu + \text {jets}}_\mathrm{{data}},&\mathcal {T} ^{\text {W}/\mathrm{t}\overline{\mathrm{t}}}_{\mu + \text {jets}} \, = \, \left( \frac{\,\mathcal {N}^{\text {W}/\mathrm{t}\overline{\mathrm{t}}}_\mathrm{{MC}}\,}{\mathcal {N}^{\mu + \text {jets}}_\mathrm{{MC}}} \right) ; \end{aligned}$$

7 $$\begin{aligned} \mathcal {N}^{\mathrm{Z}\rightarrow \nu \overline{\nu }}_\mathrm{{pred}} \,&= \, \mathcal {T} ^{{\mathrm{Z}\rightarrow \nu \overline{\nu }}}_{\mu + \text {jets}} \; \mathcal {N}^{\mu + \text {jets}}_\mathrm{{data}},&\mathcal {T} ^{{\mathrm{Z}\rightarrow \nu \overline{\nu }}}_{\mu + \text {jets}} \, = \, \left( \frac{\,\mathcal {N}^{\mathrm{Z}\rightarrow \nu \overline{\nu }}_\text {MC}\,}{\mathcal {N}^{\mu + \text {jets}}_\text {MC}} \right) ; \end{aligned}$$

8 $$\begin{aligned} \mathcal {N}^{\mathrm{Z}\rightarrow \nu \overline{\nu }}_\mathrm{{pred}} \,&= \, \mathcal {T} ^{\mathrm{Z}\rightarrow \nu \overline{\nu }}_{\mu \mu + \text {jets}} \; \mathcal {N}^{\mu \mu + \text {jets}}_\mathrm{{data}},&\mathcal {T} ^{\mathrm{Z}\rightarrow \nu \overline{\nu }}_{\mu \mu + \text {jets}} \, = \, \left( \frac{\,\mathcal {N}^{\mathrm{Z}\rightarrow \nu \overline{\nu }}_\text {MC}\,}{\mathcal {N}^{\mu \mu + \text {jets}}_\mathrm{{MC}}} \right) ; \end{aligned}$$

9 $$\begin{aligned} \mathcal {N}^{\mathrm{Z}\rightarrow \nu \overline{\nu }}_\mathrm{{pred}} \,&= \, \mathcal {T} ^{\mathrm{Z}\rightarrow \nu \overline{\nu }}_{\gamma + \text {jets}} \; \mathcal {N}^{\gamma + \text {jets}}_\mathrm{{data}},&\mathcal {T} ^{\mathrm{Z}\rightarrow \nu \overline{\nu }}_{\gamma + \text {jets}} \, = \, \left( \frac{\,\mathcal {N}^{\mathrm{Z}\rightarrow \nu \overline{\nu }}_\mathrm{{MC}}\,}{\mathcal {N}^{\gamma + \text {jets}}_\text {MC}} \right) . \end{aligned}$$

The likelihood function, described in Sect. 7, encodes the estimate via the $\mathcal {T}$ factors of the $\text {W}/\mathrm{t}\overline{\mathrm{t}}$ background, as well as the three independent estimates of the $\mathrm{Z}\rightarrow \nu \overline{\nu }$ background, which are considered simultaneously.

Several sources of uncertainty in the $\mathcal {T}$ factors are evaluated. The most relevant effects are discussed below, and generally fall into one of two categories. The first category concerns uncertainties in the “scale factor” corrections applied to simulation, which are determined using inclusive data samples that are defined by loose selection criteria, to account for the mismodelling of theoretical and experimental parameters. The second category concerns “closure tests” in data that probe various aspects of the accuracy of the simulation to model correctly the $\mathcal {T}$ factors in the phase space of this search.

The uncertainties in the $\mathcal {T}$ factors are studied for variations in scale factors related to the jet energy scale (that result in uncertainties in the $\mathcal {T}$ factors as large as $\sim$15%), the efficiency and misidentification probability of b quark jets (up to 5%), and the efficiency to identify well-reconstructed, isolated leptons (up to $\sim$5%). A 5% uncertainty in the total inelastic cross section, $\sigma _\text {in} = 69.0 \pm 3.5\,\text {mb}$ [88], is assumed and propagated through to the reweighting procedure to account for differences between the simulated measured pileup, which results in changes of up to $\sim$10%. The modelling of the transverse momentum of top quarks ($p_{\mathrm{T}} ^\mathrm{t}$) is evaluated by comparing the simulated and measured $p_{\mathrm{T}}$ spectra of reconstructed top quarks in $\mathrm{t}\overline{\mathrm{t}}$ events [89]. Simulated events are reweighted according to scale factors that decrease from a value of $\sim$1.2 to $\sim$0.7, with uncertainties of $\sim$10–20%, within the range $p_{\mathrm{T}} ^\mathrm{t} < 400\,\text {GeV}$. The systematic uncertainties in $\mathcal {T} ^{\text {W}/\mathrm{t}\overline{\mathrm{t}}}_{\mu + \text {jets}}$ arising from variations in the $p_{\mathrm{T}} ^\mathrm{t}$ scale factors are typically small (${\lesssim }$ 5%), due to the comparable phase space probed by the signal and control regions, while larger uncertainties (${\lesssim }$20%) in $\mathcal {T} ^{{\mathrm{Z}\rightarrow \nu \overline{\nu }}}_{\mu + \text {jets}}$ are observed due to the potential for significant contamination from $\mathrm{t}\overline{\mathrm{t}}$ when using $\text {W} (\rightarrow \ell \nu ) + \text {jets}$ to predict $\mathrm{Z}(\rightarrow \nu \overline{\nu }) + \text {jets}$.

The aforementioned systematic uncertainties, resulting from variations in scale factors, are summarised in Table 3, along with representative magnitudes. These sources of uncertainty are each assumed to originate from a unique underlying source and so the effect of each source is varied assuming a fully correlated behaviour across the full phase space of the signal and control regions.

The second category of uncertainty is determined from sets of closure tests based on data control samples [31]. Each set uses the observed event counts in up to eight bins in $H_{\mathrm {T}}$ for each of the nine $n_{\text {jet}}$ event categories in one of the three independent data control regions. These counts are used with the corresponding $\mathcal {T}$ factors, determined from simulation, to obtain a prediction $\mathcal {N}^\text {pred}(n_{\text {jet}}, H_{\mathrm {T}})$ of the observed yields $\mathcal {N}^\text {obs}(n_{\text {jet}}, H_{\mathrm {T}})$ in another control sample (or, in one case, $n_{\text {b}}$ event category).

Each set of tests targets a specific (potential) source of bias in the simulation modelling that may introduce an $n_{\text {jet}}$- or $H_{\mathrm {T}}$-dependent source of systematic bias in the $\mathcal {T}$ factors [31]. Several sets of tests are performed. The $\mathrm{Z}/\gamma$ ratio determined from simulation is tested against the same ratio measured using $\mathrm{Z}(\rightarrow \mu \mu ) + \text {jets}$ events and the $\gamma + \text {jets}$ sample. The $\text {W}/\mathrm{Z}$ ratio is also probed using the $\mu + \text {jets}$ and $\mu \mu + \text {jets}$ samples, which directly tests the simulation modelling of vector boson production, as well as the modelling of $\mathrm{t}\overline{\mathrm{t}}$ contamination in the $\mu + \text {jets}$ sample. A further set probes the modelling of the relative composition between $\text {W} (\rightarrow \ell \nu ) + \text {jets}$ and $\mathrm{t}\overline{\mathrm{t}}$ events using $\mu + \text {jets}$ events containing exactly zero or one more b-tagged jets, which represents a larger extrapolation in relative composition than used in the search. The effects of W polarisation are probed by using $\mu + \text {jets}$ events with a positively charged muon to predict those containing a negatively charged muon. Finally, the accuracy of the modelling of the efficiencies of the $\alpha _{\mathrm {T}}$ and $\varDelta \phi ^{*}_\mathrm{{min}}$ requirements are estimated using the $\mu + \text {jets}$ sample.

For each set of tests, the level of closure, ($\mathcal {N}^\text {obs} - \mathcal {N}^\text {pred}) / \mathcal {N}^\text {obs}$, which considers only statistical uncertainties, is inspected to ensure no statistically significant biases are observed as a function of the nine $n_{\text {jet}}$ categories or the eight $H_{\mathrm {T}}$ bins. In the absence of such a bias, the level of closure is recomputed by integrating over either all monojet and asymmetric $n_{\text {jet}}$ categories, or the symmetric $n_{\text {jet}}$ categories. The level of closure and its statistical uncertainty are combined in quadrature to determine additional contributions to the uncertainties in the $\mathcal {T}$ factors. These uncertainties are considered to be fully correlated between the monojet and asymmetric $n_{\text {jet}}$ categories or the symmetric $n_{\text {jet}}$ categories, and fully uncorrelated between these two regions in $n_{\text {jet}}$ and $H_{\mathrm {T}}$ bins. If the closure tests use the $\mu \mu + \text {jets}$ sample, the level of closure is determined by additionally integrating over pairs of adjacent $H_{\mathrm {T}}$ bins. These uncertainties, derived from the closure tests in data, are summarised in Table 3, along with representative magnitudes. These uncertainties are the dominant contribution to the total uncertainty in the $\mathcal {T}$ factors, due to the limited number of events in the data control regions.

As introduced in Sect. 5.4, templates are derived from simulation to predict the $H_{\mathrm {T}}^{\text {miss}}$ distributions of the background. The uncertainties in the $\mathcal {T}$ factors are used to constrain the normalisation of the $H_{\mathrm {T}}^{\text {miss}}$ templates. The uncertainties in the $H_{\mathrm {T}}^{\text {miss}}$ shape are discussed below.

The accuracy to which the simulation describes the $H_{\mathrm {T}}^{\text {miss}}$ distributions is evaluated with respect to data in each ($n_{\text {jet}}$, $n_{\text {b}}$, $H_{\mathrm {T}}$) bin in each of the $\mu + \text {jets}$, $\mu \mu + \text {jets}$, and $\gamma + \text {jets}$ data control regions. The level of agreement between data and simulation, defined in terms of the ratio of observed and expected counts (from simulation) as a function of $H_{\mathrm {T}}^{\text {miss}}$, is parameterised using an orthogonal first-order polynomial, $f(x) = p_0 + p_1(\bar{x}-x)$, and described by two uncorrelated parameters, $p_0$ and $p_1$. A binned likelihood fit is performed in each ($n_{\text {jet}}$, $n_{\text {b}}$, $H_{\mathrm {T}}$) bin of each control region, and the best fit value $p_1$ and its uncertainty is used to determine the presence of biases dependent on $H_{\mathrm {T}}^{\text {miss}}$. The pull of $p_1$ from a value of zero is defined as the best fit value over its standard deviation, considering only statistical uncertainties associated with the finite size of the data and simulated samples.

The lower bound of the final (open) bin in $H_{\mathrm {T}}^{\text {miss}}$ is not more than 800$\,\text {GeV}$ and is bounded from above by the upper bound of the $H_{\mathrm {T}}$ bin in question. The lower bound of the final $H_{\mathrm {T}}^{\text {miss}}$ bin is merged with lower bins if fewer than ten events in the data control regions are observed. If a bin in ($n_{\text {jet}}$, $n_{\text {b}}$, $H_{\mathrm {T}}$) contains fewer than ten events, the $H_{\mathrm {T}}^{\text {miss}}$ template is not used and the background estimates are determined inclusively with respect to $H_{\mathrm {T}}^{\text {miss}}$. The merging of bins is typically only relevant for event categories that satisfy $n_{\text {b}} \ge 2$.

**Fig. 3:**
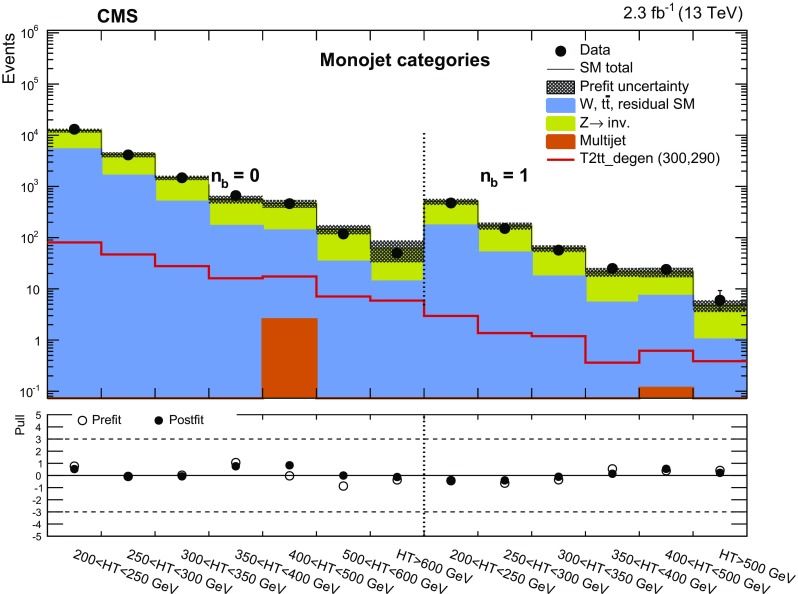
(*Upper panel*) Event yields observed in data (*solid circles*) and pre-fit SM expectations with their associated uncertainties (*black histogram with shaded band*), integrated over $H_{\mathrm {T}}^{\text {miss}}$, as a function of $n_{\text {b}}$ and $H_{\mathrm {T}}$ for the monojet category ($n_{\text {jet}} = 1$) in the signal region. For illustration only, the expectations for a benchmark model (T2tt_degen with $m_{\,\widetilde{\mathrm{t}}} = 300\,\text {GeV}$ and $m_{\widetilde{\chi }^{0}_{1}} = 290\,\text {GeV}$) are superimposed on the SM-only expectations. (*Lower panel*) The significance of deviations (pulls) observed in data with respect to the pre-fit (*open circles*) and post-fit (*closed circles*) SM expectations, expressed in terms of the total uncertainty in the SM expectations. The pulls cannot be considered independently due to inter-bin correlations

**Fig. 4:**
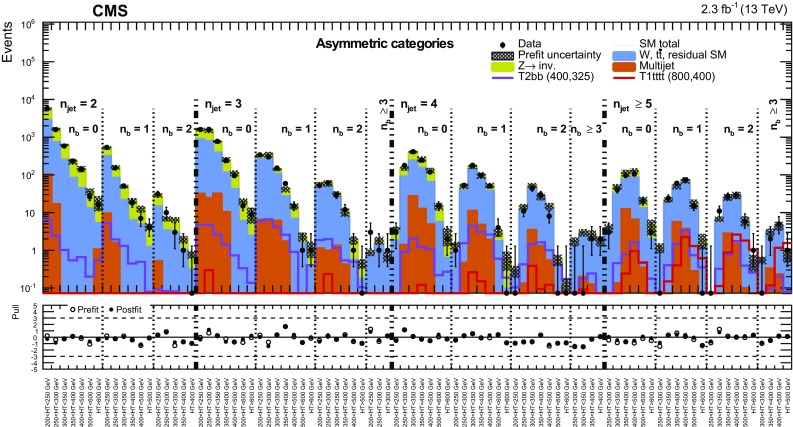
(*Upper panel*) Event yields observed in data (*solid circles*) and pre-fit SM expectations with their associated uncertainties (*black histogram with shaded band*), integrated over $H_{\mathrm {T}}^{\text {miss}}$, as a function of $n_{\text {jet}}$, $n_{\text {b}}$, and $H_{\mathrm {T}}$ for the asymmetric $n_{\text {jet}}$ categories in the signal region. For illustration only, the expectations for two benchmark models (T2bb with $m_{\,\widetilde{\mathrm{b}}} = 400\,\text {GeV}$ and $m_{\widetilde{\chi }^{0}_{1}} = 325\,\text {GeV}$, T1tttt with $m_{\,\widetilde{\mathrm{g}}} = 800\,\text {GeV}$ and $m_{\widetilde{\chi }^{0}_{1}} = 400\,\text {GeV}$) are superimposed on the SM-only expectations. (*Lower panel*) The significance of deviations (pulls) observed in data with respect to the pre-fit (*open circles*) and post-fit (*closed circles*) SM expectations, expressed in terms of the total uncertainty in the SM expectations. The pulls cannot be considered independently due to inter-bin correlations

**Fig. 5:**
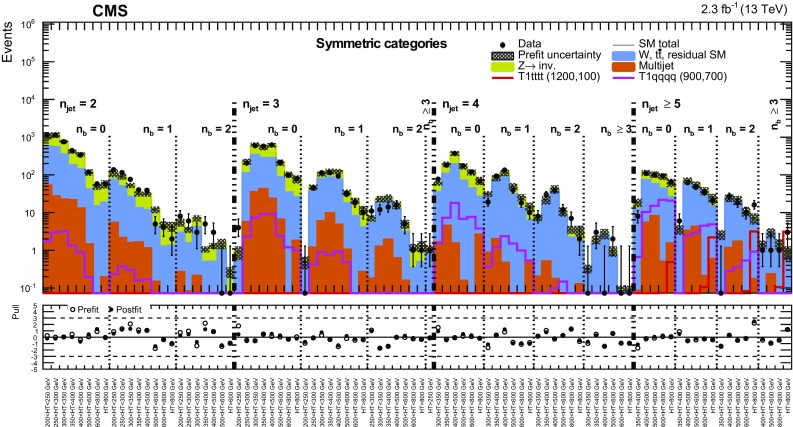
(*Upper panel*) Event yields observed in data (*solid circles*) and pre-fit SM expectations with their associated uncertainties (*black histogram with shaded band*), integrated over $H_{\mathrm {T}}^{\text {miss}}$, as a function of $n_{\text {jet}}$, $n_{\text {b}}$, and $H_{\mathrm {T}}$ for the symmetric $n_{\text {jet}}$ categories in the signal region. For illustration only, the expectations for two benchmark models (T1tttt with $m_{\,\widetilde{\mathrm{g}}} = 1200\,\text {GeV}$ and $m_{\widetilde{\chi }^{0}_{1}} = 100\,\text {GeV}$, T1qqqq with $m_{\,\widetilde{\mathrm{g}}} = 900\,\text {GeV}$ and $m_{\widetilde{\chi }^{0}_{1}} = 700\,\text {GeV}$) are superimposed on the SM-only expectations. (*Lower panel*) The significance of deviations (pulls) observed in data with respect to the pre-fit (*open circles*) and post-fit (*closed circles*) SM expectations, expressed in terms of the total uncertainty in the SM expectations. The pulls cannot be considered independently due to inter-bin correlations

**Fig. 6:**
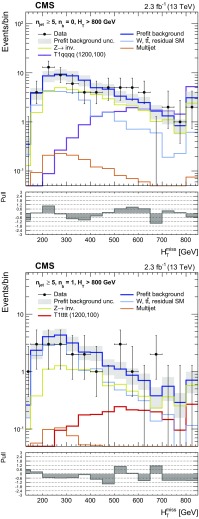
Event yields observed in data (*solid circles*) and pre-fit SM expectations with their associated uncertainties (*blue histogram with shaded band*) as a function of $H_{\mathrm {T}}^{\text {miss}}$ for events in the signal region that satisfy $n_{\text {jet}} \ge 5$, $H_{\mathrm {T}} > 800\,\text {GeV}$, and (*top*) $n_{\text {b}} = 0$ or (*bottom*) $n_{\text {b}} = 1$. For illustration only, the expectations for one of two benchmark models (T1qqqq and T1tttt, both with $m_{\,\widetilde{\mathrm{g}}} = 1200\,\text {GeV}$ and $m_{\widetilde{\chi }^{0}_{1}} = 100\,\text {GeV}$) are superimposed on the SM-only expectations. The *lower panels* indicate the significance of deviations (pulls) observed in data with respect to both the pre-fit SM expectations, expressed in terms of the total uncertainty in the SM expectations. The pulls cannot be considered independently due to inter-bin correlations

The presence of systematic biases is evaluated at a statistical level by considering the distribution of pulls obtained from each control region, which are consistent with statistical fluctuations, with no indication of trends across the full phase space of each control region. The *p*-values obtained from the fits are uniformly distributed.

The uncertainty in the $H_{\mathrm {T}}^{\text {miss}}$ modelling is extracted under the hypothesis of no bias. This is done using the maximum likelihood (ML) values of the fit parameters to determine the statistical precision to which this hypothesis can be confirmed. The quadrature sum of the ML value and its uncertainty for $p_1$ from each fit is used to define alternative templates that represent $\pm 1\sigma$ variations to the nominal $H_{\mathrm {T}}^{\text {miss}}$ template. These alternative templates are encoded in the likelihood function, as described in Sect. 7. The observed variations are compatible with the expected values obtained from studies relying only on simulated event samples. The uncertainties in the final $H_{\mathrm {T}}^{\text {miss}}$ bin of the templates depend on the event category and $H_{\mathrm {T}}$ bin, and are typically found to be in the range $\sim$10–100%.

The effect on the $H_{\mathrm {T}}^{\text {miss}}$ templates is determined under $\pm 1\sigma$ variations in the jet energy scale, the efficiency and misidentification probability of b-quark jets, the efficiency to identify well-reconstructed, isolated leptons, the pileup reweighting, and the modelling of the top quark $p_{\mathrm{T}}$. These effects are easily covered by the uncertainties determined from data, as described above, across the full phase space of the control regions, which mirror closely that of the signal region.

## Results

A model of the observations in all data samples, described by a likelihood function, is used to obtain a prediction of the SM backgrounds and to test for the presence of new-physics signals if the signal region is included in the ML fit. The observation in each bin defined by the $n_{\text {jet}}$, $n_{\text {b}}$, $H_{\mathrm {T}}$, and $H_{\mathrm {T}}^{\text {miss}}$ variables is modelled as a Poisson-distributed variable around the SM expectation and a potential signal contribution (assumed to be zero in the following discussion), where the SM expectation is the sum over the estimated contributions from all background processes according to the methods described in Sect. 6.

The nonmultijet backgrounds are related to the expected yields in the $\mu + \text {jets}$, $\mu \mu + \text {jets}$, and $\gamma + \text {jets}$ control samples via the transfer factors derived from simulation, as described in Sect. 5.5. Estimates of the contribution from multijet events in the signal region are determined according to the method described in Sect. 6.1, and are included in the likelihood function.

The systematic uncertainties summarised in Table 3 are accommodated in the likelihood function through the use of nuisance parameters, the measurements of which are assumed to follow a log-normal distribution. Alternative templates are used to describe the uncertainties in the modelling of the $H_{\mathrm {T}}^{\text {miss}}$ variable. A vertical template morphing scheme [90] is used to interpolate between the nominal and alternative $H_{\mathrm {T}}^{\text {miss}}$ templates. A nuisance parameter controls the interpolation, which is Gaussian distributed with a mean of zero and a standard deviation of one, where ±1 corresponds to the alternative templates for a ±1$\sigma$ variation in the uncertainty. Each template is interpolated quadratically between ±1$\sigma$, and a linear extrapolation is employed beyond these bounds.

The data are inspected to ascertain whether they are well described by the null (SM-only) hypothesis. This is done by considering the “pre-fit” SM background estimates, which are determined from observed data counts in the control regions only. The pre-fit result of this search is summarised in Figs. 3, 4 and 5 for, respectively, the monojet, asymmetric, and symmetric topologies. The figures also show the significance of deviations observed in data with respect to the pre-fit SM expectations expressed in terms of the total uncertainty in the SM expectations (“pull”). The data are well described by the background-only hypothesis. Figures 3, 4 and 5 also summarise the pulls from the post-fit result, which is based on a ML fit to observations in the signal region as well as the control regions.

A quantitative statement on the degree of compatibility between the observed yields and the SM expectations under the background-only hypothesis is obtained from a goodness-of-fit test based on a log likelihood ratio. The alternative hypothesis is defined by a “saturated” model [91], for which the background expectation is set equal to the observed number of events, and provides a reference for the largest value that the likelihood can take for any model for the given data set. Hence, this reference can be used as a reasonable normalisation for the maximum value observed for a more constraining model. A *p*-value of 0.20 is observed for the fit over the full signal region, and *p*-values in the range 0.04–1.00, consistent with a uniform distribution, are obtained when considering events categorised according to $n_{\text {jet}}$.

The covariance and correlation matrices for the pre-fit SM expectations in all bins of the signal region, defined by $n_{\text {jet}}$, $n_{\text {b}}$, $H_{\mathrm {T}}$, and integrated over $H_{\mathrm {T}}^{\text {miss}}$, are determined from 500 pseudo-experiments by sampling the pre-fit nuisance parameters under the background-only hypothesis. The SM expectations for different $n_{\text {jet}}$ and $n_{\text {b}}$ categories exhibit a nonnegligible level of covariance within the same $H_{\mathrm {T}}$ bin, primarily as a result of the systematic uncertainties evaluated from closure tests, described in Sect. 6.2, that integrate yields over $n_{\text {jet}}$ and $n_{\text {b}}$. Bins adjacent and next-to-adjacent in $n_{\text {jet}}$ and/or $n_{\text {b}}$ can have correlation coefficients in the range 0.2–0.4, and, infrequently, as large as $\sim$0.5. Otherwise, the correlation coefficients are <0.2. Anticorrelation coefficients are typically not larger than $\sim$0.2.

Figure 6 shows the event yields observed in data and pre-fit SM expectations with their associated uncertainties as a function of $H_{\mathrm {T}}^{\text {miss}}$ for two categories of candidate signal events, which provide good sensitivity to models with high-mass gluinos. For illustration only, the expected counts from benchmark signal models that assume the pair production and decay of gluinos, described further in Sect. 8, are also shown.

## Interpretation

### Specification for simplified models

The results of the search are used to constrain simplified SUSY models [92–94]. Each model assumes the pair production of gluinos or squarks and their subsequent prompt decays to SM particles and the LSP with a 100% branching fraction (unless indicated otherwise). The gluino decays contain intermediate on-shell SUSY particle states (such as the top squark or the chargino) for a subset of the models. All other SUSY particles are assumed to be too heavy ($m_{\widetilde{\mathrm{g}}} / m_{\widetilde{\mathrm{q}}} = 10\,\text {TeV}$) to be produced directly. Three-body decays of gluinos are assumed to occur via off-shell squarks of light or heavy flavour. Off-shell decays are processed by pythia in a single three- or four-body step, without taking into account the width or polarisation of the parent: this is true for the top-squark four-body decay ($\widetilde{\mathrm{t}} \rightarrow \mathrm{{bf}}\overline{\mathrm{{f}}}'\widetilde{\chi }^{0}_{1}$), as well as the three-body decay of the chargino ($\widetilde{\chi }^\pm _1 \rightarrow \text {f}\bar{\text {f}}'\widetilde{\chi }^{0}_{1}$), where f and f$'$ are fermions produced in the decay of an intermediate off-shell W boson.

Fourteen unique production and decay modes are considered, which yield a range of topologies and final states (with only the all-jet final state considered in this search). Each class of simplified model is identified by a label that indicates the topology and final state, and scans in the gluino or squark ($m_{\widetilde{\mathrm{g}}} / m_{\widetilde{\mathrm{q}}}$) and LSP ($m_{\widetilde{\chi }^{0}_{1}}$) mass parameter space are performed. Table 4 summarises the production and decay modes, as well as any additional assumptions that define the simplified models. The models can be categorised according to the following descriptions: the gluino-mediated and direct production of light-flavour squarks, the gluino-mediated production of off-shell third-generation squarks, the natural gluino-mediated production of on-shell top squarks, and the direct production of on-shell third-generation squarks. In the case of direct pair production of light-flavour squarks, two different assumptions on the theoretical production cross section are made. For the “eightfold” scenario (T2qq_8fold), the scalar partners to left- and right-handed quarks of the u, d, s, and c flavours are assumed to be light and degenerate in mass, with other squark states decoupled to a high mass. For the “onefold” scenario (T2qq_1fold), only a single light squark is assumed to participate in the interaction and all other squarks are decoupled to a high mass.

Under the signal$+$background hypothesis, and in the presence of a nonzero signal contribution, a modified frequentist approach is used to determine upper limits at the 95% confidence level (CL) on the cross section, $\sigma _\mathrm{{UL}}$, to produce pairs of SUSY particles as a function of the parent SUSY particle and the LSP masses. The limits can be expressed in terms of the signal strength parameter, $\mu$, which is determined relative to the theoretical cross section that is calculated at NLO $+$ NLL accuracy. An Asimov data set [95] is used to determine the expected upper limit on the allowed cross section for a given model. The potential contributions from a new-physics signal to each of the signal and control regions are considered, even though the only significant contribution occurs in the signal region and not in the control regions (i.e. signal contamination). The approach is based on the one-sided (so called LHC-style) profile likelihood ratio as the test statistic [96] and the $\mathrm {CL}_\mathrm {s}$ criterion [97, 98]. Asymptotic formulae [95] are utilised to approximate the distributions of the test statistics under the SM background-only and signal+background hypotheses.

### Acceptances and uncertainties

The experimental acceptance times efficiency ($\mathcal {A}\,\varepsilon$) and its uncertainty are evaluated independently for each model class as a function of ($m_\text {SUSY}, m_\text {LSP}$). Table 5 summarises $\mathcal {A}\,\varepsilon$ for a number of benchmark models for which the search yields an expected exclusion ($\mu \lesssim 1$). For each topology, typically two different pairs of parent SUSY particle and LSP masses ($m_\text {SUSY}, m_\text {LSP}$) are chosen that are characterised by a large and a small (i.e. compressed) difference in parent SUSY particle and LSP masses. The four most sensitive event categories, defined in terms of $n_{\text {jet}}$, are used to determine $\sigma _\text {UL}$. The categories used per benchmark model are listed in Table 5, along with $\mathcal {A}\,\varepsilon$ determined for these four categories.

The effects of several sources of uncertainty on $\mathcal {A}\,\varepsilon$, as well as the potential for event migration between bins of the signal region, are considered. The potential effect of each source of uncertainty is assessed by including in the likelihood function the alternative normalisations and shapes for the $H_{\mathrm {T}}^{\text {miss}}$ templates with respect to the nominal versions. The nominal and alternative templates are obtained from simulated event samples for the signal models, and the alternative templates effectively propagate the various input uncertainties to determine their effects on the $n_{\text {jet}}$, $n_{\text {b}}$, $H_{\mathrm {T}}$, and $H_{\mathrm {T}}^{\text {miss}}$ distributions for the signal model.

In addition to the uncertainty in the integrated luminosity of 2.7% [99], the following sources of uncertainty are dominant: the statistical uncertainty arising from the finite size of simulated signal samples, the modelling of ISR, the jet energy corrections (JEC) evaluated in simulation, and the modelling of scale factors applied to simulated event samples that correct for differences in the efficiency and misidentification probability of b-quark jets ($\text {SF}_\text {b-tag}$). The magnitude of each contribution depends on the model and the masses of the parent SUSY particle and LSP.

**Table 4:** A summary of the simplified SUSY models used to interpret the results of this search. All on-shell SUSY particles in the decay are stated

Model class	Production	Decay	Additional assumptions
Gluino-mediated and direct production of light-flavour squarks			
T1qqqq	$\mathrm {p}\mathrm {p}\rightarrow \widetilde{\mathrm{g}} \widetilde{\mathrm{g}}$	$\widetilde{\mathrm{g}} \rightarrow \overline{\mathrm{q}}\mathrm{q}\widetilde{\chi }^{0}_{1}$	–
T2qq_8fold	$\mathrm {p}\mathrm {p}\rightarrow \widetilde{\mathrm{q}} \overline{\widetilde{\mathrm{q}}}$	$\widetilde{\mathrm{q}} \rightarrow \mathrm{q}\widetilde{\chi }^{0}_{1}$	$m_{\widetilde{\mathrm{q}}} = m_{\widetilde{\mathrm{q}} _\mathrm{L}} = m_{\widetilde{\mathrm{q}} _\mathrm{R}}$, $\widetilde{\mathrm{q}} = \{ \widetilde{\mathrm{u}}, \widetilde{\mathrm{d}}, \widetilde{\mathrm{s}}, \widetilde{\mathrm{c}} \}$
T2qq_1fold	$\mathrm {p}\mathrm {p}\rightarrow \widetilde{\mathrm{q}} \overline{\widetilde{\mathrm{q}}}$	$\widetilde{\mathrm{q}} \rightarrow \mathrm{q}\widetilde{\chi }^{0}_{1}$	$m_{\widetilde{\mathrm{q}} (\widetilde{\mathrm{q}} \ne \widetilde{\mathrm{u}} _\mathrm{L})} \gg m_{\widetilde{\mathrm{u}} _\mathrm{L}}$
Gluino-mediated production of off-shell third-generation squarks			
T1bbbb	$\mathrm {p}\mathrm {p}\rightarrow \widetilde{\mathrm{g}} \widetilde{\mathrm{g}}$	$\widetilde{\mathrm{g}} \rightarrow \overline{\mathrm{b}}\mathrm{b}\widetilde{\chi }^{0}_{1}$	–
T1tttt	$\mathrm {p}\mathrm {p}\rightarrow \widetilde{\mathrm{g}} \widetilde{\mathrm{g}}$	$\widetilde{\mathrm{g}} \rightarrow \overline{\mathrm{t}}\widetilde{\mathrm{t}} ^*\rightarrow \overline{\mathrm{t}}\mathrm{t}\widetilde{\chi }^{0}_{1}$	–
T1ttbb	$\mathrm {p}\mathrm {p}\rightarrow \widetilde{\mathrm{g}} \widetilde{\mathrm{g}}$	$\widetilde{\mathrm{g}} \rightarrow \overline{\mathrm{t}}\mathrm{b}\widetilde{\chi }^\pm _1\rightarrow \overline{\mathrm{t}}\mathrm{b}\text {W} ^*\widetilde{\chi }^{0}_{1}$	$m_{\widetilde{\chi }^\pm _1} - m_{\widetilde{\chi }^{0}_{1}} = 5\,\text {GeV}$
Natural gluino-mediated production of on-shell top squarks			
T5tttt_DM175	$\mathrm {p}\mathrm {p}\rightarrow \widetilde{\mathrm{g}} \widetilde{\mathrm{g}}$	$\widetilde{\mathrm{g}} \rightarrow \overline{\mathrm{t}}\widetilde{\mathrm{t}} \rightarrow \overline{\mathrm{t}}\mathrm{t}\widetilde{\chi }^{0}_{1}$	$m_{\,\widetilde{\mathrm{t}}} - m_{\widetilde{\chi }^{0}_{1}} = 175\,\text {GeV}$
T5ttcc	$\mathrm {p}\mathrm {p}\rightarrow \widetilde{\mathrm{g}} \widetilde{\mathrm{g}}$	$\widetilde{\mathrm{g}} \rightarrow \overline{\mathrm{t}}\widetilde{\mathrm{t}} \rightarrow \overline{\mathrm{t}}\mathrm{c}\widetilde{\chi }^{0}_{1}$	$m_{\,\widetilde{\mathrm{t}}} - m_{\widetilde{\chi }^{0}_{1}} = 20\,\text {GeV}$
Direct production of on-shell third-generation squarks			
T2bb	$\mathrm {p}\mathrm {p}\rightarrow \widetilde{\mathrm{b}} \overline{\widetilde{\mathrm{b}}}$	$\widetilde{\mathrm{b}} \rightarrow \mathrm{b}\widetilde{\chi }^{0}_{1}$	–
T2tb	$\mathrm {p}\mathrm {p}\rightarrow \widetilde{\mathrm{t}} \overline{\widetilde{\mathrm{t}}}$	$\widetilde{\mathrm{t}} \rightarrow \mathrm{t}\widetilde{\chi }^{0}_{1} \;\text {or}\; \mathrm{b}\widetilde{\chi }^\pm _1\rightarrow \mathrm{b}\text {W} ^*\widetilde{\chi }^{0}_{1}$	$50/50\%$, $m_{\widetilde{\chi }^\pm _1} - m_{\widetilde{\chi }^{0}_{1}} = 5\,\text {GeV}$
T2tt	$\mathrm {p}\mathrm {p}\rightarrow \widetilde{\mathrm{t}} \overline{\widetilde{\mathrm{t}}}$	$\widetilde{\mathrm{t}} \rightarrow \mathrm{t}\widetilde{\chi }^{0}_{1}$	–
T2cc	$\mathrm {p}\mathrm {p}\rightarrow \widetilde{\mathrm{t}} \overline{\widetilde{\mathrm{t}}}$	$\widetilde{\mathrm{t}} \rightarrow \mathrm{c}\widetilde{\chi }^{0}_{1}$	$10< m_{\,\widetilde{\mathrm{t}}} - m_{\widetilde{\chi }^{0}_{1}} < 80\,\text {GeV}$
T2tt_degen	$\mathrm {p}\mathrm {p}\rightarrow \widetilde{\mathrm{t}} \overline{\widetilde{\mathrm{t}}}$	$\widetilde{\mathrm{t}} \rightarrow \mathrm{b}\text {W} ^*\widetilde{\chi }^{0}_{1}$	$10< m_{\,\widetilde{\mathrm{t}}} - m_{\widetilde{\chi }^{0}_{1}} < 80\,\text {GeV}$
T2tt_mixed	$\mathrm {p}\mathrm {p}\rightarrow \widetilde{\mathrm{t}} \overline{\widetilde{\mathrm{t}}}$	$\widetilde{\mathrm{t}} \rightarrow \mathrm{c}\widetilde{\chi }^{0}_{1} \;\text {or}\; \mathrm{b}\text {W} ^*\widetilde{\chi }^{0}_{1}$	$50/50\%$, $10< m_{\,\widetilde{\mathrm{t}}} - m_{\widetilde{\chi }^{0}_{1}} < 80\,\text {GeV}$

**Table 5:** A summary of benchmark simplified models, the most sensitive $n_{\mathrm{jet}}$ categories categories, and representative values for the corresponding experimental acceptance times efficiency ($\mathcal {A}\,\varepsilon$), the dominant systematic uncertainties, the theoretical production cross section ($\sigma _\text {theory}$), and the expected and observed upper limits on the production cross section, expressed in terms of the signal strength parameter ($\mu$)

Benchmark models		Most sensitive	$\mathcal {A}$ $\varepsilon$	Systematic uncertainties [%]				$\sigma _{\mathrm{theory}}$	$\mu$ (95% CL)	
(m$_{\mathrm{SUSY}}$, m$_{\mathrm{LSP}})$ [GeV]		n$_{\mathrm{jet}}$ categories	[%]	MC stat.	ISR	JEC	SF$_{\mathrm{b-tag}}$	[fb]	Exp.	Obs.
Tlqqqq	(1300, 100)	$\ge$5, 4, 3, 2	21.2	7–30	$\sim$2	4–21	2–14	46.1	0.79	0.76
	(900, 700)	$\ge$5, $\ge$5a, 4, 4a	12.8	10–33	1–13	1–26	1–10	677	0.58	0.44
T2qq_8fold	(1050, 100)	$\ge$5, 3, 4, 2	40.3	7–33	1–4	3–16	1–11	35.2	0.90	0.63
	(650, 550)	$\ge$5, 4, $\ge$5a, 4a	6.3	10–28	1–16	2–29	1–6	864	0.93	0.80
T2qq_1fold	(600, 50)	5, 3, 2, 4	30.2	5–33	1–5	1–30	1–8	177	0.78	0.84
	(400, 250)	$\ge$5, 4, $\ge$5a, 3	7.1	8–30	1–8	3–25	1–7	1849	0.73	0.71
T1bbbb	(1500, 100)	$\ge$5, 4, 3, 2	22.7	5–17	1–2	1–12	2–22	14.2	0.81	0.79
	(1000, 800)	$\ge$5, 4, $\ge$5a, 4a	11.4	8–31	1–17	1–40	1–14	325	0.33	0.32
T1ttbb	(1300, 100)	$\ge$5, $\ge$5a, 4, 3	5.3	7–16	1–2	2–7	2–12	46.1	1.00	1.89
	(800, 400)	$\ge$5, $\ge$5a, 4, 4a	1.5	7–27	1–2	3–45	1–8	1490	0.56	1.03
T1ttbb	(1300, 100)	$\ge$5, 4, 3, $\ge$5a	8.5	9–32	1–2	3–16	2–19	46.1	0.60	0.91
	(1000, 700)	$\ge$5, $\ge$5a, 4, 3	7.7	9–30	1–9	3–65	1–14	325	0.51	0.70
T5tttt_DM175	(800, 100)	$\ge$5, $\ge$5a, 3, 4	0.5	12–20	2–4	3–5	1–6	1490	0.69	1.19
	(700, 400)	$\ge$5, $\ge$5a, 4, 4a	0.5	20–29	2–10	8–10	1–2	3530	1.00	1.35
T5ttcc	(1200, 200)	$\ge$5, 4, 3, $\ge$5a	11.0	6–25	5–25	3–21	1–24	85.6	0.58	0.87
	(750, 600)	$\ge$5, $\ge$5a, 4, 4a	2.2	9–23	1–4	5–21	1–3	2270	0.89	0.72
T2bb	(800, 50)	2, 3, 4, $\ge$5	34.9	5–31	2–6	1–21	1–23	28.3	0.96	1.06
	(375, 300)	$\ge$5, 4, 3a, 3	3.2	8–33	1–10	3–25	1–7	2610	0.67	0.87
T2tb	(600, 50)	$\ge$5, 4, 3, 2	13.4	3–28	1–3	1–22	1–17	175	0.70	1.35
	(350, 225)	$\ge$5, 4, 3, 3a	2.3	9–33	1–4	2–41	1–8	3790	0.79	0.88
T2tt	(700, 50)	$\ge$5, 4, 3, $\ge$5a	18.2	8–33	1–4	2–22	1–21	67.0	0.90	1.19
	(350, 100)	$\ge$5, $\ge$5a, 4a, 4	3.4	7–31	1–1	1–28	1–7	3790	0.44	0.50
T2cc	(325, 305)	$\ge$5, 4, 3, 2	1.9	3–32	1–27	1–27	1–12	5600	0.92	0.68
T2tt_deqen	(300, 290)	3,4, $\ge$5, 2	2.0	2–27	1–27	1–25	1–12	8520	0.56	0.41
T2tt_mixed	(300, 250)	$\ge$5, 4, $\ge$5a, 4a	1.0	3–33	1–27	1–33	1–13	8520	0.99	0.58

The $\mathcal {A}\,\varepsilon$ for models with small mass splittings (e.g. $m_{\widetilde{\mathrm{q}}} - m_{\widetilde{\chi }^{0}_{1}} \lesssim m_{\text{ t }}$) is due largely to ISR, the modelling of which is evaluated by comparing the simulated and measured $p_{\mathrm{T}}$ spectra of the system recoiling against the ISR jets in $\mathrm{t}\overline{\mathrm{t}}$ events, using the technique described in Ref. [100]. The uncertainty can be as large as $\sim$30%, and is the dominant systematic uncertainty for systems with a compressed mass spectrum. Uncertainties in the jet energy scale, as large as $\sim$40%, can also be dominant for models characterised by high jet multiplicities in the final state. The uncertainties in $\text {SF}_\text {b-tag}$ can be as large as $\sim$25%. Table 5 summarises these dominant contributions to the uncertainty in $\mathcal {A}\,\varepsilon$ for a range of benchmark models. Characteristic values for each model are expressed in terms of a range that is representative of the values across all bins of the signal region. The upper bound for each range may be subject to moderate statistical fluctuations.

Further uncertainties with subdominant contributions are considered on a similar footing. The uncertainties in the efficiency of identifying well-reconstructed, isolated leptons are considered, with a typical magnitude of $\sim$5% and treated as anticorrelated between the signal and control regions. The uncertainty of 5% in $\sigma _\text {in}$ is propagated through to the reweighting procedure to account for differences between the simulated and measured pileup. Finally, uncertainties in the simulation modelling of the efficiencies of the trigger strategy employed by the search are typically <10%.

The choice of PDF set, or variations therein, predominantly affects $\mathcal {A}\,\varepsilon$ through changes in the $p_{\mathrm{T}}$ spectrum of the system recoil, which is covered by the ISR uncertainty, hence no additional uncertainty is adopted. Uncertainties in $\mathcal {A}\,\varepsilon$ due to variations in the renormalisation and factorisation scales are determined to be $\sim$5%. In both cases, contributions to the uncertainty in the theoretical production cross section are considered.

### Cross section and mass exclusions

Limits for each of the aforementioned benchmark models are summarised in Table 5, expressed in terms of $\mu$. All benchmark models are expected to be excluded. The observed limits fluctuate around the expected $\mu$ values, with some models exhibiting a moderately weaker than expected limit.

**Fig. 7:**
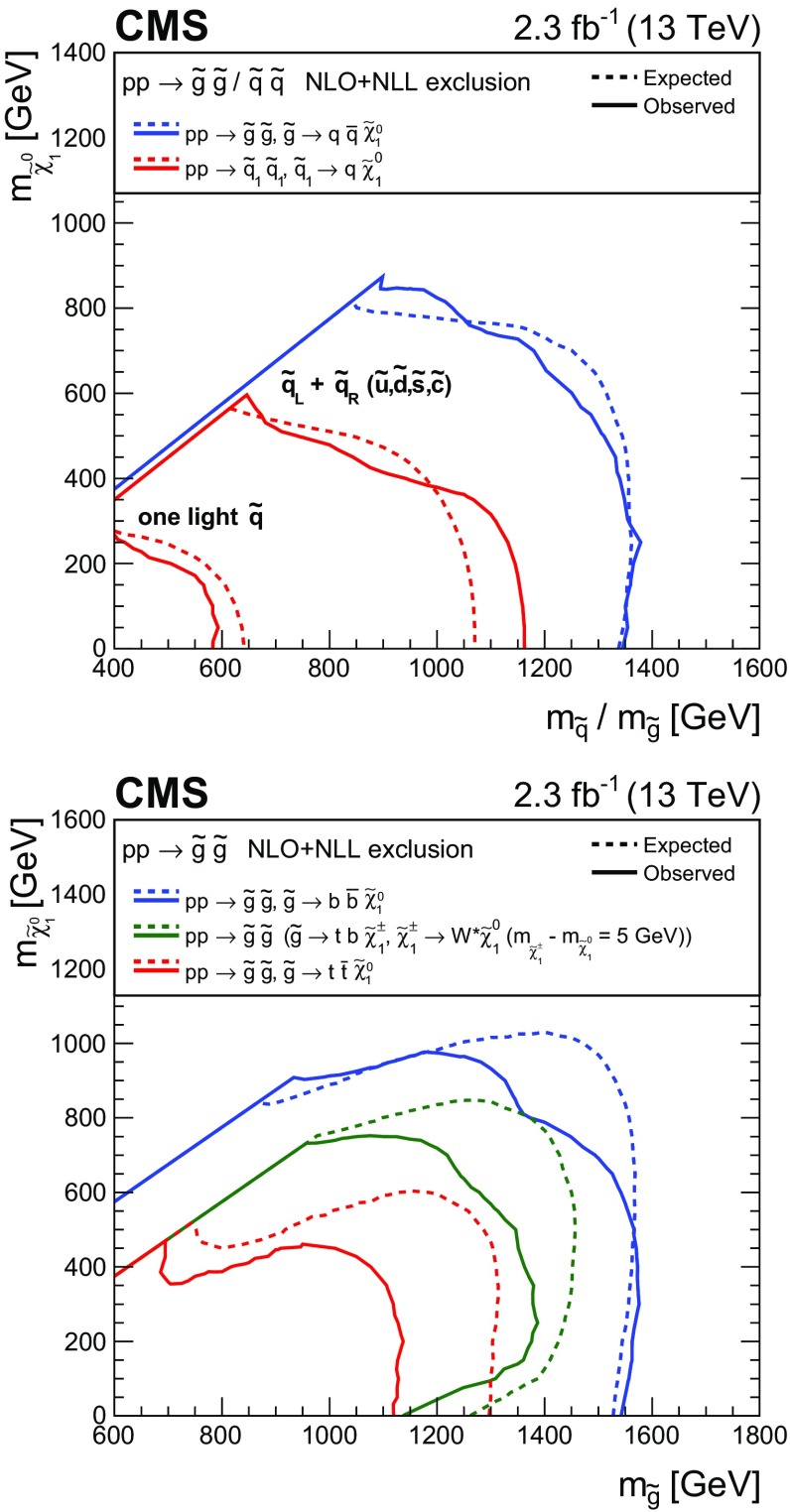
Observed and expected mass exclusions at 95% CL (indicated, respectively, by *solid* and *dashed contours*) for various classes of simplified models. (*Top*) Gluino-mediated or direct pair production of light-flavour squarks. The two scenarios involve, respectively, the decay $\widetilde{\mathrm{g}} \rightarrow \overline{\mathrm{q}}\mathrm{q}\widetilde{\chi }^{0}_{1}$ (T1qqqq) and $\widetilde{\mathrm{q}} \rightarrow \mathrm{q}\widetilde{\chi }^{0}_{1}$, and the latter involves two assumptions on the mass degeneracy of the squarks (T2qq_8fold and T2qq_1fold). (*Bottom*) Three scenarios involving the gluino-mediated pair production of off-shell third-generation squarks: $\widetilde{\mathrm{g}} \rightarrow \overline{\mathrm{b}}\mathrm{b}\widetilde{\chi }^{0}_{1}$ (T1bbbb), $\widetilde{\mathrm{g}} \rightarrow \overline{\mathrm{t}}\widetilde{\mathrm{t}} ^*\rightarrow \overline{\mathrm{t}}\mathrm{t}\widetilde{\chi }^{0}_{1}$ (T1tttt), and $\widetilde{\mathrm{g}} \rightarrow \overline{\mathrm{t}}\mathrm{b}\widetilde{\chi }^\pm _1\rightarrow \overline{\mathrm{t}}\mathrm{b}\text {W} ^*\widetilde{\chi }^{0}_{1}$ (T1ttbb)

**Fig. 8:**
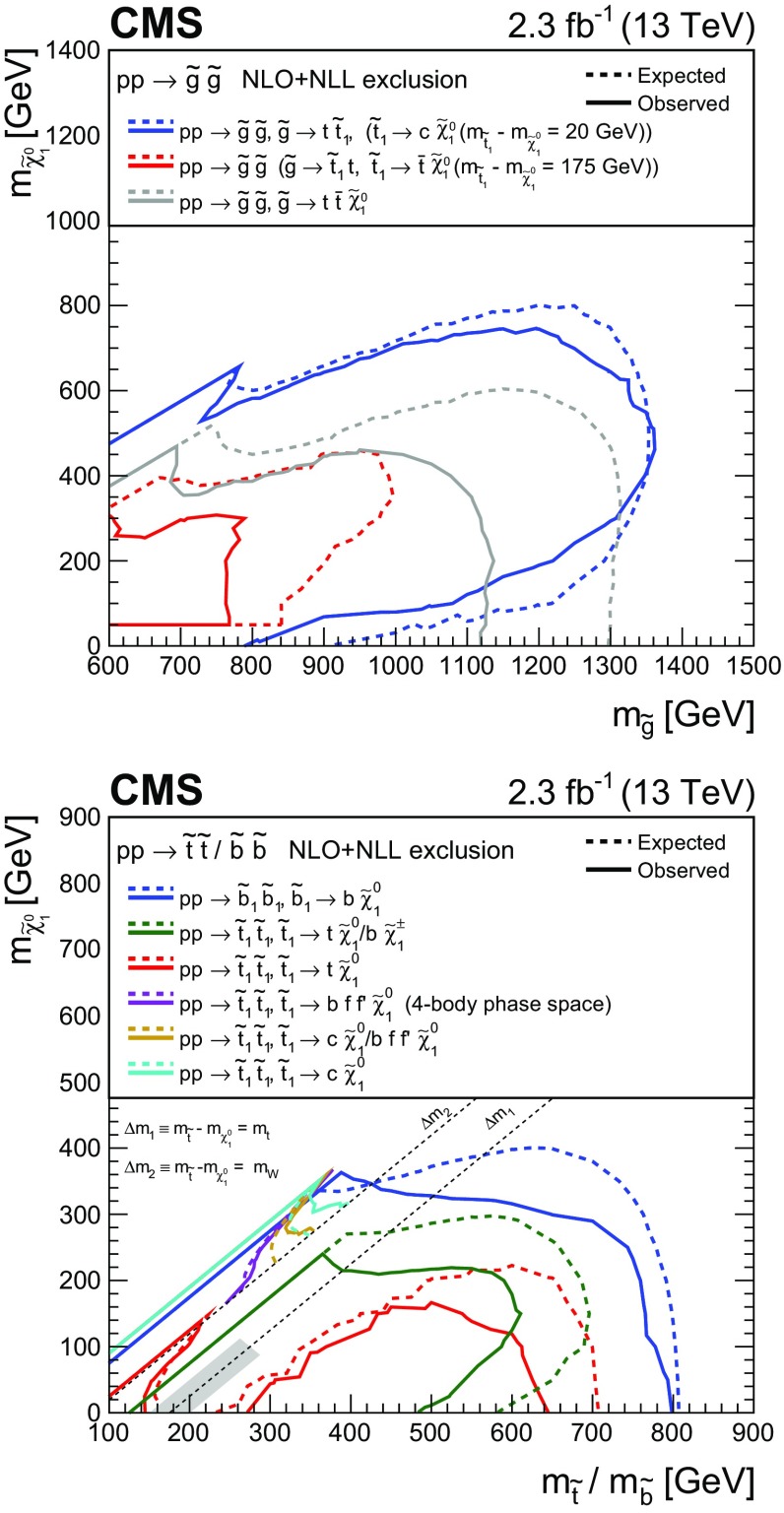
Observed and expected mass exclusions at 95% CL (indicated, respectively, by *solid* and *dashed contours*) for a number of simplified models. (*Top*) Two scenarios involving the gluino-mediated pair production of on-shell top squarks: $\widetilde{\mathrm{g}} \rightarrow \overline{\mathrm{t}}\widetilde{\mathrm{t}} \rightarrow \overline{\mathrm{t}}\mathrm{t}\widetilde{\chi }^{0}_{1}$ with $m_{\,\widetilde{\mathrm{t}}} - m_{\widetilde{\chi }^{0}_{1}} = 175\,\text {GeV}$ (T5tttt_DM175) and $\widetilde{\mathrm{g}} \rightarrow \overline{\mathrm{t}}\widetilde{\mathrm{t}} \rightarrow \overline{\mathrm{t}}\mathrm{c}\widetilde{\chi }^{0}_{1}$ with $m_{\,\widetilde{\mathrm{t}}} - m_{\widetilde{\chi }^{0}_{1}} = 20\,\text {GeV}$ (T5ttcc). Also shown, for comparison, is T1tttt. (*Bottom*) Six scenarios involving the direct pair production of third-generation squarks. The first scenario involves the pair production of bottom squarks, $\widetilde{\mathrm{b}} \rightarrow \mathrm{b}\widetilde{\chi }^{0}_{1}$ (T2bb). Two scenarios involve the decay of top squark pairs as follows: $\widetilde{\mathrm{t}} \rightarrow \mathrm{t}\widetilde{\chi }^{0}_{1}$ or $\widetilde{\mathrm{t}} \rightarrow \mathrm{b}\widetilde{\chi }^\pm _1\rightarrow \mathrm{b}\text {W} ^*\widetilde{\chi }^{0}_{1}$ with $m_{\widetilde{\chi }^\pm _1} - m_{\widetilde{\chi }^{0}_{1}} = 5\,\text {GeV}$ and branching fractions $50/50\%$ (T2tb), or $\widetilde{\mathrm{t}} \rightarrow \mathrm{t}\widetilde{\chi }^{0}_{1}$ (T2tt). The final three scenarios consider top squark decays under the assumption $10< m_{\,\widetilde{\mathrm{t}}} - m_{\widetilde{\chi }^{0}_{1}} < 80\,\text {GeV}$: $\widetilde{\mathrm{t}} \rightarrow \mathrm{c}\widetilde{\chi }^{0}_{1}$ (T2cc), $\widetilde{\mathrm{t}} \rightarrow \mathrm{b}\text {W} ^*\widetilde{\chi }^{0}_{1}$ (T2tt_degen), and $\widetilde{\mathrm{t}} \rightarrow \mathrm{c}\widetilde{\chi }^{0}_{1}$ or $\widetilde{\mathrm{t}} \rightarrow \mathrm{b}\text {W} ^*\widetilde{\chi }^{0}_{1}$ with branching fractions $50/50\%$ (T2tt_mixed). The *grey shaded region* denotes T2tt models that are not considered for interpretation

Figures 7 and 8 summarise the disfavoured regions of the mass parameter space for the fourteen classes of simplified models. These regions are derived by comparing the upper limits on the measured fiducial cross section, corrected for the experimental $\mathcal {A}\,\varepsilon$, with the theoretical cross sections calculated at NLO+NLL accuracy in $\alpha _\mathrm{{s}}$ [33]. The former cross section value is determined as a function of $m_{\widetilde{\mathrm{g}}}$ or $m_{\widetilde{\mathrm{q}}}$ and $m_{\widetilde{\chi }^{0}_{1}}$, while the latter has a dependence solely on $m_{\widetilde{\mathrm{g}}}$ or $m_{\widetilde{\mathrm{q}}}$. The exclusion of models is evaluated using observed data counts in the signal region (solid contours) and also expected counts based on an Asimov data set (dashed contours).

Figure 7 (upper) shows the excluded mass parameter space for models that assume the gluino-mediated or direct production of light-flavour squarks. The excluded region extends to higher masses for the gluino-mediated production of light-flavour squarks (T1qqqq), with respect to direct pair production when assuming an eightfold degeneracy in mass (T2qq_8fold), due to a combination of a higher gluino pair production cross section and a final state characterised by higher jet multiplicities, which are exploited to provide better signal-to-background separation. The excluded mass region is significantly reduced when assuming only a single light squark (T2qq_1fold), with limits weakening due to the lower production cross section, compounded by the reduced signal-to-background ratios achieved in the core of distributions in the discriminating variables.

Figure 7 (lower) shows the exclusion contours for models that assume the gluino-mediated pair production of off-shell third-generation squarks. For the topologies T1tttt and T1bbbb, each gluino is assumed to undergo a three-body decay via, respectively, an off-shell top or bottom squark to a quark-antiquark pair of the same flavour and the $\widetilde{\chi }^{0}_{1}$. In the case of T1ttbb, each gluino is assumed to undergo a three-body decay to an on-shell chargino, $\widetilde{\chi }^\pm _1$, a bottom quark, and a top antiquark. The chargino mass is defined relative to the neutralino mass via the expression $m_{\widetilde{\chi }^\pm _1} - m_{\widetilde{\chi }^{0}_{1}} = 5\,\text {GeV}$. The chargino decays promptly to the $\widetilde{\chi }^{0}_{1}$ and an off-shell W boson. The excluded mass regions differ significantly for these topologies, primarily due to the different number of (on-shell) W bosons in their final states, resulting in the highest $\mathcal {A} \, \varepsilon$ for T1bbbb and lowest for T1tttt. Further, $\mathcal {A} \, \varepsilon$ has a strong dependence on jet multiplicity, which is highest for T1tttt, due to the $\varDelta \phi ^{*}_\mathrm{{min}}$ variable. An additional feature for T1ttbb is the weakening of the mass limit at low values of $m_{\widetilde{\chi }^{0}_{1}}$, when $m_{\widetilde{\chi }^\pm _1} = m_{\widetilde{\chi }^{0}_{1}} + 5\,\text {GeV} \lesssim m_\text {t}$. In this scenario, the $\widetilde{\chi }^\pm _1$ (and hence $\widetilde{\chi }^{0}_{1}$) is not highly Lorentz boosted relative to the top quark resulting from the three-body decay of the gluino. Hence, two $\widetilde{\chi }^{0}_{1}$ SUSY particles do not carry away significant ${\vec {p}}_{\mathrm {T}}^{\text {miss}}$, which is instead realised through W boson decays to neutrinos and “lost” leptons or $\tau$ leptons that decay to neutrinos and hadrons. The observed mass limits for these topologies are up to $\sim$2 standard deviations weaker than the expected limits. These differences are due to upward fluctuations in data for two contiguous bins that satisfy the requirements $n_{\text {jet}} \ge 5$, $n_{\text {b}} \ge 2$, and $H_{\mathrm {T}} > 800\,\text {GeV}$. This region has the highest sensitivity to models involving gluino production and decays to third-generation quarks (via on- or off-shell squarks). The observed counts are consistent with statistical fluctuations and the events do not exhibit anomalous nonphysical behaviour. The events are distributed in $H_{\mathrm {T}}^{\text {miss}}$ consistent with expectation, hence models characterised by high values of $H_{\mathrm {T}}^{\text {miss}}$, such as T1bbbb with $m_{\widetilde{\mathrm{g}}} \gg m_{\widetilde{\chi }^{0}_{1}}$ or $m_{\widetilde{\mathrm{g}}} \approx m_{\widetilde{\chi }^{0}_{1}}$, are less compatible with the data counts in this high-$n_{\text {jet}}$, $n_{\text {b}}$, and $H_{\mathrm {T}}$ region.

Figure 8 (upper) shows exclusion contours for models that assume gluino pair production, with each gluino decaying to a top quark and an on-shell top squark, the latter of which decays to SM particles and the LSP. As discussed earlier, these models can be considered as representations of a natural solution to the little hierarchy problem. Two different scenarios are considered for the decay of the top squarks. The T5tttt_DM175 models assume a two-body decay to a top quark and the $\widetilde{\chi }^{0}_{1}$, with the top squark mass defined relative to the $\widetilde{\chi }^{0}_{1}$ as $m_{\widetilde{\mathrm{t}}} - m_{\widetilde{\chi }^{0}_{1}} = m_\text {t}$. Models that satisfy $m_{\widetilde{\chi }^{0}_{1}} < 50\,\text {GeV}$ are not considered here, as the $\widetilde{\chi }^{0}_{1}$ particles carry very little momentum. The T5ttcc models assume $m_{\widetilde{\mathrm{t}}} - m_{\widetilde{\chi }^{0}_{1}} = 20\,\text {GeV}$ and two-body decays to a charm quark and the $\widetilde{\chi }^{0}_{1}$.

**Table 6:** Summary of the mass limits obtained for the fourteen classes of simplified models. The limits indicate the strongest observed and expected (in parentheses) mass exclusions in $\widetilde{\mathrm{g}}$, $\widetilde{\mathrm{q}}$, $\widetilde{\mathrm{b}}$, $\widetilde{\mathrm{t}}$, and $\widetilde{\chi }^{0}_{1}$. The quoted values have uncertainties of ±25 and ±10$\,\text {GeV}$ for models involving the pair production of, respectively, gluinos and squarks

Model class	Parent SUSY particle	Best mass limit [$\text {GeV}$ ]	
		$\widetilde{\mathrm{g}}/\widetilde{\mathrm{q}}/\widetilde{\mathrm{b}}/\widetilde{\mathrm{t}}$	$\widetilde{\chi }^{0}_{1}$
T1qqqq	$\widetilde{\mathrm{g}}$	1375 (1350)	875 (850)
T2qq_8fold	$\widetilde{\mathrm{q}}$	1150 (1075)	600 (550)
T2qq_1fold	$\widetilde{\mathrm{q}}$	575 (650)	275 (275)
T1bbbb	$\widetilde{\mathrm{g}}$	1575 (1575)	975 (1025)
T1tttt	$\widetilde{\mathrm{g}}$	1125 (1325)	475 (600)
T1ttbb	$\widetilde{\mathrm{g}}$	1375 (1450)	750 (850)
T5tttt_DM175	$\widetilde{\mathrm{g}}$	800 (1000)	300 (450)
T5ttcc	$\widetilde{\mathrm{g}}$	1350 (1350)	700 (800)
T2bb	$\widetilde{\mathrm{b}}$	800 (800)	360 (400)
T2tb	$\widetilde{\mathrm{t}}$	610 (690)	240 (300)
T2tt (3-body)	$\widetilde{\mathrm{t}}$	670 (720)	210 (240)
T2tt (2-body)	$\widetilde{\mathrm{t}}$	280 (280)	200 (200)
T2cc	$\widetilde{\mathrm{t}}$	400 (350)	310 (340)
T2tt_degen	$\widetilde{\mathrm{t}}$	370 (360)	360 (350)
T2tt_mixed	$\widetilde{\mathrm{t}}$	360 (350)	350 (340)

Finally, Fig. 8 (lower) shows exclusion contours for models that assume the direct production of pairs of third-generation squarks. For the T2bb models, bottom squarks are pair produced and each decays to a bottom quark and the $\widetilde{\chi }^{0}_{1}$. The T2tt models assume top squarks are pair produced and each is assumed to undergo a two- or three-body decay to, respectively, a top quark and the $\widetilde{\chi }^{0}_{1}$ when $m_{\widetilde{\mathrm{t}}} - m_{\widetilde{\chi }^{0}_{1}} > m_\text {t}$ is satisfied, or a b quark, an on-shell W boson, and the $\widetilde{\chi }^{0}_{1}$ for the condition $m_{\text {W}}< m_{\widetilde{\mathrm{t}}} - m_{\widetilde{\chi }^{0}_{1}} < m_\text {t}$. Models that satisfy $|m_{\widetilde{\mathrm{t}}} - m_\text {t} - m_{\widetilde{\chi }^{0}_{1}} | < 25\,\text {GeV}$ and $m_{\widetilde{\mathrm{t}}} + m_{\widetilde{\chi }^{0}_{1}} < 375\,\text {GeV}$ are not considered here, as $\sigma _\text {UL}$ is a strong function of $m_{\widetilde{\mathrm{t}}} - m_{\widetilde{\chi }^{0}_{1}}$ in this low-$m_{\widetilde{\mathrm{t}}}$ region due to the high levels of signal contamination found in the $\mu + \text {jets}$ control region for models that resemble the $\mathrm{t}\overline{\mathrm{t}}$ background in terms of their topological and kinematic properties. The T2tb models also assume the pair production of top squarks, with each undergoing a two-body decay to either a top quark and the $\widetilde{\chi }^{0}_{1}$, or a bottom quark and the $\widetilde{\chi }^\pm _1$, with equal branching fractions of 50%. As for the T1ttbb models, the chargino mass is defined relative to the neutralino mass via the expression $m_{\widetilde{\chi }^\pm _1} - m_{\widetilde{\chi }^{0}_{1}} = 5\,\text {GeV}$, and the chargino decays promptly to the $\widetilde{\chi }^{0}_{1}$ and an off-shell W boson. The excluded mass regions differ significantly for the T2bb, T2tb, and T2tt topologies, in an analogous way to the T1bbbb, T1ttbb, and T1tttt models described above. The difference in the mass exclusions is due primarily to the different number of (on-shell) W bosons in the final states, which affects $\mathcal {A} \, \varepsilon$ through the presence of leptons from the decay of the W boson. An additional feature for T2tb is the weakening of the mass limit at low values of $m_{\widetilde{\chi }^{0}_{1}}$, when $m_{\widetilde{\chi }^\pm _1} = m_{\widetilde{\chi }^{0}_{1}} + 5\,\text {GeV} \lesssim m_\text {t}$. Moderately weaker than expected mass limits are observed for all models involving two-body decays, which is traced to mild upward fluctuations in data for events satisfying $n_{\text {jet}} = 2$, $n_{\text {b}} = 2$, and $350< H_{\mathrm {T}} < 500\,\text {GeV}$.

Figure 8 (lower) also shows exclusion contours for models that assume the pair production of top squarks but a near-mass-degenerate system that satisfies $10\,\text {GeV}< m_{\widetilde{\mathrm{t}}} - m_{\widetilde{\chi }^{0}_{1}} < m_\text {W}$. Two decays of the top squark are considered. The T2cc and T2tt_degen models assume two- and four-body decays of the top squark to, respectively, a charm quark and the $\widetilde{\chi }^{0}_{1}$, or to $\text {bf}\bar{\text {f}}'\widetilde{\chi }^{0}_{1}$, where $\text {f}$ and $\bar{\text {f}}'$ are fermions produced in the decay of an intermediate off-shell W boson. A third class of models, T2tt_mixed, assumes both these decay modes with an equal branching fraction of 50%. For T2cc, the excluded mass region is relatively stable as a function of the mass splitting $\Delta m = m_{\widetilde{\mathrm{t}}} - m_{\widetilde{\chi }^{0}_{1}}$, with $\widetilde{\mathrm{t}}$ masses excluded up to 400$\,\text {GeV}$. For T2tt_degen, the excluded mass region is strongly dependent on $\Delta m$, weakening considerably for increasing values of $\Delta m$ due to the increased momentum phase space available to leptons produced in the four-body decay. The T2tt_mixed models exhibit an intermediate behaviour. Mass limits for all three model classes converge for the smallest mass splitting considered, $\Delta m = 10\,\text {GeV}$, when the SM particles from the $\widetilde{\mathrm{t}}$ decay are extremely soft and outside the experimental acceptance. An approximately contiguous mass exclusion limit is observed across the transition from the T2tt_degen four-body to the T2tt three-body decay of the $\widetilde{\mathrm{t}}$, as the top quark moves on-shell. The excluded mass region weakens further as $\Delta m \rightarrow m_\text {t}$.

Table 6 summarises the strongest expected and observed mass limits for each class of simplified model.

## Summary

An inclusive search for new-physics phenomena is reported, based on data from pp collisions at $\sqrt{s} = 13\,\text {TeV}$. The data are recorded with the CMS detector and correspond to an integrated luminosity of $2.3 \pm 0.1 {\,\text {fb}^{-1}}$. The final states analysed contain one or more jets with large transverse momenta and a significant imbalance of transverse momentum, as expected from the production of massive coloured SUSY particles, each decaying to SM particles and the lightest stable, weakly-interacting, SUSY particle.

The sums of the standard model backgrounds are estimated from a simultaneous binned likelihood fit to the observed yields for samples of events categorised according to the number of reconstructed jets, the number of jets identified as originating from b quarks, and the scalar and the magnitude of the vector sums of the transverse momenta of jets. In addition to the signal region, $\mu + \text {jets}$, $\mu \mu + \text {jets}$, and $\gamma + \text {jets}$ control regions are included in the likelihood fit. The observed yields are found to be in agreement with the expected contributions from standard model processes. The search result is interpreted in the mass parameter space of fourteen simplified SUSY models, which cover scenarios that involve the gluino-mediated or direct production of light- or heavy-flavour squarks, intermediate SUSY particle states, as well as natural and nearly mass-degenerate spectra.

The increase in the centre-of-mass energy of the LHC, from 8 to 13$\,\text {TeV}$, provides a significant gain in sensitivity to heavy particle states such as gluinos. In the case of pair-produced gluinos, each decaying via an off-shell b squark to the b quark and the LSP, models with masses up to $\sim$1.6 and $\sim$1.0$\,\text {TeV}$ are excluded for, respectively, the gluino and LSP. These limits improve on those obtained at $\sqrt{s} = 8\,\text {TeV}$ by, respectively, $\sim$250 and $\sim$300$\,\text {GeV}$. In the case of direct pair production, models with masses up to $\sim$800 and $\sim$350$\,\text {GeV}$ are excluded for, respectively, the b squark and LSP. These mass limits are sensitive to the assumptions on the squark flavour and the presence of intermediate states such as charginos.

Finally, a comprehensive study of nearly mass-degenerate models involving top squark pair production is performed. The two decay modes of the top squark are the loop-induced two-body decay to the neutralino and one c quark, and the four-body decay to the neutralino, one b quark, and an off-shell W boson. A third scenario is considered in which the two modes are simultaneously open, each with a branching fraction of 50%. Masses of the top squark and LSP up to, respectively, 400 and 360$\,\text {GeV}$ are excluded, depending on the decay modes considered.

In conclusion, the analysis provides sensitivity across a large region of the natural SUSY parameter space, as characterised by interpretations with several simplified models. In particular, these studies improve on existing limits for nearly mass-degenerate models involving the production of pairs of top squarks.
